# Scoring Functions for Protein-Ligand Binding Affinity Prediction Using Structure-based Deep Learning: A Review

**DOI:** 10.3389/fbinf.2022.885983

**Published:** 2022-06-17

**Authors:** Rocco Meli, Garrett M. Morris, Philip C. Biggin

**Affiliations:** ^1^ Department of Biochemistry, University of Oxford, Oxford, United Kingdom; ^2^ Department of Statistics, University of Oxford, Oxford, United Kingdom

**Keywords:** drug discovery, scoring function, artificial intelligence, explainable AI, deep learning, neural network, affinity

## Abstract

The rapid and accurate *in silico* prediction of protein-ligand binding free energies or binding affinities has the potential to transform drug discovery. In recent years, there has been a rapid growth of interest in deep learning methods for the prediction of protein-ligand binding affinities based on the structural information of protein-ligand complexes. These structure-based scoring functions often obtain better results than classical scoring functions when applied within their applicability domain. Here we review structure-based scoring functions for binding affinity prediction based on deep learning, focussing on different types of architectures, featurization strategies, data sets, methods for training and evaluation, and the role of explainable artificial intelligence in building useful models for real drug-discovery applications.

## 1 Introduction

The discovery and development of new small-molecule drugs is a very challenging and expensive process (Drews 2000; Dickson and Gagnon 2004; Schneider and Schneider 2016). Only a handful of new drugs are approved each year (Brown and Wobst 2021), which is minuscule compared to the vastness of chemical space (Reymond et al., 2010) and the billions of dollars poured into drug discovery campaigns (DiMasi et al., 2016). The discovery pipeline for small-molecule drugs usually starts with the identification of a protein target against which a hit compound is identified by high throughput screening (HTS) (Mayr and Bojanic 2009; Macarron et al., 2011). The hit compound is subsequently optimized to obtain a lead compound with good potency and favorable pharmacodynamics and pharmacokinetics properties.

Thanks to significant methodological and hardware advances, computer-aided drug discovery (CADD) has played an important role in the development of new small-molecule drugs over the last decades (Sliwoski et al., 2013). CADD speeds up the early stages of the drug discovery process—hit identification and hit-to-lead optimization—and lowers the costs of these phases by reducing time and experimental resources needed. CADD methods fall into two broad classes: (explicit) structure-based, and ligand-based (or implicit structure-based) methods. For the latter, similarities to known active molecules play an important role since either the protein target is unknown, or information about the protein target is either unavailable or not included. For structure-based methods, the target structure is known and this additional information is exploited in the modelling and optimization of drug-target interactions (DTIs).

One of the main goals in the computational elucidation of DTIs is the calculation of relative or absolute binding free energies to distinguish potent binders from weak binders (or non-binders) against a target of interest. A fast and accurate prediction of protein-ligand binding affinities would circumvent the need for many time-consuming and complex experiments. Rigorous computational methods based on all-atom molecular dynamics simulations in explicit solvent—such as free energy perturbation and thermodynamic integration (Adcock and McCammon 2006)—can compute accurate relative and absolute binding free energies (Bash et al., 1987; Boresch et al., 2003; Mobley et al., 2007; Aldeghi et al., 2016, Aldeghi et al., 2018a; Cournia et al., 2017), predict ligand selectivity (Aldeghi et al., 2017) and mutation effects (Aldeghi et al., 2018b; Hauser et al., 2018), and guide fragment elaborations (Alibay et al., 2022). Unfortunately, such rigorous methods are computationally expensive and often require a lot of expert knowledge and domain expertise (Mey et al., 2020; Hahn et al., 2021). This remains true even for simpler methods such as ligand-interaction energy (LIE) (Åqvist et al., 1994; Jones-Hertzog and Jorgensen 1997). Methods treating the solvent implicitly, such as the Poisson-Boltzmann and generalized Born models (Genheden and Ryde 2015), can offer significant speed increase but sometimes at the expense of accuracy.

The great successes of deep learning (DL) in the fields of computer vision (Voulodimos et al., 2018), natural language processing (NLP) (Young et al., 2018), and other fields of computer science in recent years kick-started the research and application of deep learning in many scientific disciplines including physics, chemistry, biology, and medicine (Baldi 2021). In the field of drug discovery, machine learning (ML) has been in use for a long time, and the potential usefulness of the use of deep learning in virtual screening was identified early on (Unterthiner et al., 2014). The application of modern deep learning architectures to all stages of the drug discovery pipeline is a very active area of research today (Jing et al., 2018; Brown, 2020; Muratov et al., 2020; Jiménez-Luna et al., 2021a; Gaudelet et al., 2021). The main applications in small-molecule drug design consists in the prediction of DTIs, identification of binding sites (Jiménez et al., 2017; Pu et al., 2019; Aggarwal et al., 2021), the generation of novel molecular entities (Schneider and Clark 2019; Meyers et al., 2021), and the prediction of absorption, distribution, metabolism, excretion, and toxicity (ADMET) properties (Huang D. Z. et al., 2021).

Bioactivity prediction can be performed as a classification task—where binders/actives are distinguished from non-binders/inactives—or as a regression task. Machine learning and deep learning scoring functions (SFs) for the prediction of binding affinities (regression) are useful in lead optimization, in contrast with SFs that try to identify binders amongst a large pool of non-binders (classification) and are used in virtual screening to identify a hit. Another task where SFs are commonly used is pose prediction, where near-native poses are distinguished from incorrect poses (classification). Pose prediction and binding affinity prediction are complementary tasks in molecular docking, where a pose is generated and subsequently scored according to the predicted binding affinity.

In this review, we will focus on SFs for binding affinity prediction (inhibition constant *K*_*i*_ or dissociation constant *K*_*d*_) or binding free energy prediction, but we will inevitably mention related SFs used in pose prediction and virtual screening—which often share the same algorithms and ideas. Recent reviews of structure-based SFs and deep learning for virtual screening are given by Li et al. (2021b), Kimber et al. (2021), and Rifaioglu et al. (2019). Additionally, to narrow the scope of the review, we focus on structure-based deep-learning methods and we refer the reader interested in ligand-based methods to Tropsha (2010), Muratov et al. (2020), Baskin (2020), and Palazzesi and Pozzan (2022). More general and broad reviews about the application of machine learning and deep learning in drug discovery are provided by Chen H. et al. (2018), Vamathevan et al. (2019), and Schneider et al. (2019).

## 2 Classical Scoring Functions

Historically, SFs for binding affinity prediction and virtual screening have been classified into three categories: force-field-based, empirical, and knowledge-based (Muegge and Rarey 2001; Böhm and Stahl 2002). However, recently Liu and Wang (2015) argued that this historical classification overlooks more recent developments in the field and thus proposed an updated classification scheme with four classes of scoring functions: force-field-based or physics-based, empirical or regression-based, knowledge-based or potential of mean force-based, and descriptor-based or machine learning-based.

This classification is useful to distinguish different methodologies and ideas appearing in the development of SFs. However, some SFs can’t be precisely assigned to only one category and the boundary between the four different classes remains rather fuzzy.

In this section we will briefly discuss the first three classes of SFs, often termed “classical” SFs. A good overview of the different SFs can be found in the paper of Liu and Wang (2015)—which proposed the current classification of SFs—and a more recent overview of different SFs used in protein-ligand docking is provided by Li et al. (2019a). While classical scoring functions are still actively developed and refined today, the research focus has certainly shifted to ML/DL based scoring functions.

### 2.1 Physics-Based (Force-Field Based) Scoring Functions

Physics-based (or force-field-based) SFs use energy terms of a molecular mechanics force-field—whose parameters are determined to reproduce experimental observables or *ab initio* quantum mechanical calculations (Monticelli and Tieleman 2012)—to evaluate protein-ligand interactions. The non-covalent interaction energy between protein and ligand atoms is usually expressed as the sum of van der Waals and electrostatic interaction terms. In their simplest form, such pairwise interactions are represented by a Lennard-Jones potential and Coulomb interaction between point charges.

Different physics-based scoring functions use different potentials to describe van der Waals and electrostatic interactions, depending on the design of the underlying force field. For example, the dielectric constant can be distance-dependent to take into account electrostatic screening due to the solvent and the lower dielectric constant in protein-ligand binding sites (Hingerty et al., 1985; Gilson and Honig 1986; Huang et al., 2010).

Often, additional shorter-range (and sometimes directional) terms are added to account for hydrogen bonding as well as solvation energy and therefore physics-based scoring function can take the following form:

$$\Delta G_{\text{binding}}=\Delta E_{\text{VdW}}+\Delta E_{\text{el}}+\Delta E_{\text{H}-\text{bond}}+\Delta G_{\text{sol}}.$$ (1)

The solvation energy term can take into account both polar and non-polar contributions. The former accounts for the loss of polar interactions between charged groups and water, while the latter accounts for the desolvation of hydrophobic groups upon binding.

Finally, empirical terms accounting for the loss of torsional degrees of freedom upon complexation can also be included. Oftentimes, simple approximations based on the number of rotatable bonds are used (Böhm 1994; Chang et al., 2007; Huey et al., 2007; Huang and Zou 2010), although more advanced treatments have been suggested (Guedes et al., 2021b). The same corrections are applied to empirical and knowledge-based scoring functions, discussed below.

Force-field-based scoring functions are attractive because of their physical origin and because they can leverage advances in force-field developments, including the latest advances in ML force-fields (Unke et al., 2021). However, describing solvent effects in ligand binding remains an outstanding challenge (Limongelli et al., 2012; Ross et al., 2012; Darby et al., 2019).

Notable examples of physics-based (force field-based) scoring functions are DOCK (DesJarlais et al., 1988; Meng et al., 1992; Shoichet et al., 1992; Ewing et al., 2001; Moustakas et al., 2006; Allen et al., 2015), AutoDock (Goodsell and Olson 1990) and AutoDock 2 (Morris et al., 1996) (AutoDock 3 and AutoDock 4 use hybrid scoring functions (Morris et al., 1998; Huey et al., 2007; Morris et al., 2009)), GoldScore (Jones et al., 1995, Jones et al., 1997), and GalaxyDock (Shin and Seok 2012; Shin et al., 2013).

### 2.2 Empirical (Regression-Based) Scoring Functions

Empirical or regression-based scoring functions are based on regression analysis to determine the coefficient of different pre-defined terms based on experimental data. This is also what machine learning (or descriptor-based) scoring functions do, however in empirical or regression-based scoring functions the functional form of the scoring function is predetermined and it is often quite simple (such as a linear combination of different contributions) (Ain et al., 2015). As we mentioned previously, the line between the four different classes of scoring functions suggested by Li et al. (2019a) is sometimes blurry.

Empirical scoring functions assuming a linear functional form take the following form (Guedes et al., 2018):

$$\Delta G_{\text{binding}}=w_{0}+w_{1}\Delta G_{\text{VdW}}+w_{2}\Delta G_{\text{H}-\text{bond}}+w_{3}\Delta G_{\text{entropy}}.$$ (2)

The functional form of empirical scoring functions is similar to physics-based scoring functions. However, in empirical scoring functions the parameters **w** are determined by regression analysis—usually multivariate linear regression or partial least squares (Li et al., 2019a)—to reproduce experimentally determined values.

Often, the different terms in empirical scoring functions are simple reward or penalty scores. For example, the ChemScore (Eldridge et al., 1997; Verdonk et al., 2003) scoring function has the following functional form:

$$\text{ChemScore}=w_{0}+w_{1}S_{\text{hbond}}+w_{2}S_{\text{metal}}+w_{3}S_{\text{lipo}}+w_{4}H_{\text{rot}}+E_{\text{int}}+E_{\text{clash}}+E_{\text{cov}}$$ (3)

where *S*_hbond_ is the score assigned to hydrogen bonds, *S*_metal_ scores acceptor-metal interactions, *S*_lipo_ scores lipophilic interactions, *H*_rot_ describes the loss in conformational entropy upon complexation, *E*_int_ is the ligand’s internal energy, *E*_cov_ is the covalent energy term, and *E*_clash_ represents the energetic penalty of clashes between protein and ligand atoms.

One of the first empirical scoring functions was introduced by Böhm (1994) and notable examples include ChemScore (Eldridge et al., 1997; Verdonk et al., 2003), X-Score (Wang et al., 2002), Glide (Friesner et al., 2004, 2006) DockThor (de Magalhães et al., 2014), SFCscore (Sotriffer et al., 2008). More recent scoring functions are Vinardo (Quiroga and Villarreal 2016), Lin_F9 (Yang and Zhang 2021), DockTScore (Guedes et al., 2021a) (combined with ML), and AA-Score (Pan et al., 2022).

A fairly recent review of empirical scoring functions for structure-based virtual screening is provided by Guedes et al. (2018).

### 2.3 Knowledge-Based (Potential-Based) Scoring Functions

Knowledge-based or potential-based scoring functions are based on pairwise statistical potentials of the form:

$$S=\underset{i\in \text{lig}}{\sum }\underset{j\in \text{prot}}{\sum }\omega_{ij}\left(r\right),$$ (4)

where the distance-dependent pairwise potential *ω*_*ij*_(*r*) is given by:

$$\omega_{ij}\left(r\right)=-k_{b}T\ln\left(\frac{\rho_{ij}\left(r\right)}{\rho_{ij}^{0}}s\right).$$ (5)

*ρ*_*ij*_(*r*) is the number density of pairs of type *i*-*j* at distance *r* while $\rho_{ij}^{0}$ is the same quantity for a reference state where there is no interaction between types *i* and *j* (Muegge and Martin 1999). Therefore, if *ρ*_*ij*_(*r*) is larger than the reference state $\rho_{ij}^{0}$ it contributes favorably to the scoring function while if *ρ*_*ij*_(*r*) is smaller than the reference state $\rho_{ij}^{0}$ then it contributes unfavorably to the scoring function. The pairwise potentials *ω*_*ij*_(*r*) are obtained from the analysis of interactions in a large data set of protein-ligand complexes and usually, only pairs of protein and ligand atoms within a certain cutoff are considered (*r* < *r*_cutoff_).

One of the advantages of knowledge-based scoring functions is that entropic and solvation contributions are taken into account implicitly (Muegge and Martin 1999). However, some knowledge-based scoring functions include solvation and entropy effects explicitly (Huang and Zou 2010).

Notable examples of knowledge-based (potential-based) scoring function are the SMoG (DeWitte and Shakhnovich 1996; DeWitte et al., 1997) (later extended to a hybrid knowledge-based and empirical scoring function (Debroise et al., 2017a)), the PMF scoring function developed by Muegge and co-workers (Muegge and Martin 1999; Muegge 2000, Muegge 2001), DrugScore (Gohlke et al., 2000; Velec et al., 2005; Neudert and Klebe 2011; Dittrich et al., 2018), ITScore (Huang and Zou 2006a; Huang and Zou 2006b, Huang and Zou 2010), KECSA (Zheng and Merz 2013), and M-score (Yang et al., 2005). More recent knowledge-based scoring functions are SMoG 2016 (Debroise et al., 2017b), Convex-PL (Kadukova and Grudinin 2017), DLIGAND2 (Chen P. et al., 2019), and KORP-PL (Kadukova et al., 2021).

## 3 Data Sets

To train ML and DL SFs, high-quality and reasonably large data sets are essential. The success of supervised machine learning and deep learning algorithms strongly depends on the quality and the size of the data set used for training. Thanks to the advances in high-throughput X-ray crystallography and cryo-electron microscopy (cryoEM), the number of available high-resolution structures in the Protein Data Bank (PDB) is constantly increasing (Goodsell et al., 2019b).

In this section, we briefly discuss some of the most common data sets encountered in the training and evaluation of machine learning and deep learning structure-based SFs for binding affinity prediction. The main data sets providing both co-crystal structures and experimental binding affinities are listed in Table 1.

**TABLE 1:** Main data sets providing protein-ligand complexes (crystal structures) and corresponding binding affinities. *N* is the number of protein-ligand complexes (co-crystal structures) with associated binding affinities.

Data Set	*N*	Superset	Website
PDBbind 2020	19 443	—	pdbbind.org.cn
CASF-2016	285	PDBbind 2016	pdbbind.org.cn
CASF-2013	195	PDBbind 2013	pdbbind.org.cn
CASF-2007	195	PDBbind 2007	pdbbind.org.cn
Binding MOAD 2020	15 223	—	bindingmoad.org
CSAR-NCS HiQ	343	Binding MOAD + PDBbind	csardock.org
CSAR-NCS HiQ Update	123	Binding MOAD + PDBbind	csardock.org
Astex Diverse Set	74	—	doi.org/10.1021/jm061277y
BindingDB	11 442	—	bindingdb.org
D3R GC 4	20	—	drugdesigndata.org
D3R GC 3	24	—	drugdesigndata.org
D3R GC 2	36	—	drugdesigndata.org
D3R GC 2015	24	—	drugdesigndata.org

### 3.1 PDBbind

The PDBbind dataset (Wang et al., 2004) is a curated subset of the PDB and it is arguably one of the most common data sets used to train ML and DL SFs for protein-ligand binding affinity prediction. The dataset also contains protein-protein and ligand-nucleic acid complexes.

The origin of the database can be traced back to 2004, when Wang et al. (2004) collected protein-ligand complexes from the PDB (release 103, January 2003) and screened the primary references of the identified complexes to extract binding affinity data (*K*_*d*_, *K*_*i*_, IC_50_).

To train ML and DL SFs, high-quality data is essential—although it has been demonstrated that including lower quality data can improve performance (Li et al., 2015; Francoeur et al., 2020). The PDBbind database is therefore split into a “refined” set and a “general” set (Wang et al., 2004, Wang et al., 2005). The “refined” set is a selection of protein-ligand crystal structures with a resolution of 2.5 Å or lower, where there is a single ligand that is non-covalently bound without significant steric clashes (Wang et al., 2005). Only systems with associated equilibrium constants *K*_*i*_ and *K*_*d*_ are included in the refined set—IC_50_ values depend on the design of the binding assay—and complexes are filtered to only contain common organic elements.

The same approach was used to build the PDBbind refined set version 2007 (Cheng et al., 2009), but it was improved to produce the PDBbind refined set 2013 and subsequent versions (Li et al., 2014b; Liu et al., 2014, 2017). In addition to the previous criteria used to compile the PDBbind refined set 2007, the complexes added to the PDBbind refined set 2013 satisfy the following additional criteria (Li et al., 2014b): no missing backbone or side chain fragments within 8 Å from the ligand, no extreme values of binding affinity (1 pm < *K* < 10 mm, where *K* = {*K*_*i*_, *K*_*d*_}), no multiple binding sites with significantly different binding affinities ( >10 folds difference), no non-standard amino acids within 5 Å from the ligand, and no shallow binders ( <15% of buried ligand surface). The rules for selecting protein-ligand complexes into the PDBbind refined set 2013, together with their rationale and the difference with the rules used for the PDBbind refined set 2007, are very clearly summarized by Li et al. (2014b).

The PDBbind dataset can be downloaded from pdbbind.org.cn. The current release (PDBbind 2020) collects binding affinities and structural data for 23 496 biomolecular complexes, 19 443 of which are protein-ligand complexes.

### 3.1.1 CASF

The CASF benchmarks are a series of comparative assessments of scoring functions originally introduced by Cheng et al. (2009). They evaluate different scoring functions for their performance on scoring, ranking, docking, and screening on a diverse and high-quality set of protein-ligand complexes. Originally employed to compare mostly classical SFs, it has become the *de facto* standard for an initial evaluation of ML and DL SFs (especially for protein-ligand binding affinity prediction).

To test different scoring functions on a diverse and high-quality data set of protein-ligand complexes, a data set is extracted from the PDBbind refined set (where high-quality complexes have already been identified). The PDBbind refined set is clustered according to sequence similarity using BLAST (Altschul et al., 1990), with a similarity threshold of 90% (Cheng et al., 2009). This means that proteins with a sequence similarity higher than 90% are collected in the same cluster since they are likely to represent the same protein or the same protein family.

Once proteins from the PDBbind refined set are clustered by sequence similarity, clusters containing at least four complexes are retained (Cheng et al., 2009). This results in a total of 65 clusters, from which three complexes are sampled: the complex with lower binding affinity, the complex with higher binding affinity, and the complex with binding affinity closer to the mean between the highest and lowest binding affinities (Cheng et al., 2009). This clustered sub-sampling of the PDBbind refined set (called PDBbind core set) results in a total of 65 × 3 = 195 protein-ligand complexes used for the first comparative assessment of scoring functions (CASF-2007).

For the CASF-2013 comparative assessment of scoring functions (Li et al., 2014a), the construction of the PDBbind core set was improved by using the same sequence similarity program used by the PDB, and only clusters with five (and not four) proteins were retained (Li et al., 2014b). Additionally, the best binding affinity has to differ at least 10-fold from the median binding affinity, and the median binding affinity has to differ at least 10-fold from the poorest binding affinity (Li et al., 2014b). The electron density maps of the remaining complexes were visually assessed; if a complex failed at this step, the next best candidate was selected amongst the same cluster (Li et al., 2014b). The final PDBbind core set 2013 still consists of 195 protein-ligand complexes from 65 protein clusters (Li et al., 2014b).

The core set for CASF-2016 (Su et al., 2018) brought additional refinements and more data. As usual, the systems within the high-quality benchmark set are selected from the 4057 protein-ligand complexes in the PDBbind refined set (version 2016). The clustering of complexes based on protein sequence similarity remains the same. However, for CASF-2016, five representatives of each cluster were selected instead of the three selected for CASF-2007 and CASF-2013 (Su et al., 2018). The representative complexes were selected according to their binding affinity: the complex with the lowest binding affinity, the complex with the highest binding affinity, and three complexes distributed as evenly as possible between the lowest and highest binding affinity (Su et al., 2018). The lowest and highest binding affinities differ at least 100-fold and the difference between consecutive binding affinities is at least 1-fold. All ligands were inspected to ensure that there are no identical ligands or stereoisomers (Su et al., 2018). The final PDBbind core set (CASF-2016 benchmark set) consists of 57 × 5 = 285 protein-ligand complexes and it is arguably one of the test sets encountered more frequently in the development of ML and DL SFs.

Unlike the PDBbind data set, the CASF benchmark is not updated annually and therefore the latest release to date remains CASF-2016. The CASF benchmark packages can be downloaded from pdbbind.org.cn/casf.php.

It is very common for ML and DL SFs to be trained on the PDBbind refined or general set and subsequently tested on the CASF benchmark set. Recently, non-redundant subsets of the PDBbind refined set were introduced by Boyles et al. (2019) and Su et al. (2020) to evaluate the ability of ML and DL SFs to generalize when removing increasingly dissimilar examples from the training set that have some similarities with the CASF benchmark set.

### 3.2 Binding MOAD

The Binding MOAD (Mother Of All Databases) (Hu et al., 2005; Benson et al., 2007; Ahmed et al., 2014; Smith et al., 2019) is a subset of the PDB that collects high-quality and biologically relevant crystal structures of protein-ligand complexes together with experimentally determined binding affinities. Ligands available in the Binding MOAD include small peptides (ten amino acids or less), small oligonucleotides (four nucleotides or fewer), small and drug-like organic molecules, and enzymatic cofactors. Crystal structures have a resolution better than 2.5 Å. As for the PDBbind data set, experimental binding affinities are collected from the primary reference of the deposited PDB structure and consists of only *K*_*i*_, *K*_*d*_ or IC_50_ values.

The Binding MOAD was first introduced in 2005, containing 5331 protein-ligand complexes from 1780 unique protein families and 2630 unique ligands (Hu et al., 2005). 1375 protein-ligand complexes were associated with binding affinity data spanning 13 orders of magnitude (Hu et al., 2005). The 1780 unique protein families were used to create a non-redundant subset for which 475 complexes have binding affinity data (Hu et al., 2005).

The Binding MOAD is extracted from the PDB as follows (Hu et al., 2005). The full PDB database is screened for high-resolution structures (better than 2.5 Å) excluding theoretical models and NMR structures. Structures containing nucleic acids larger than four nucleotides and peptides longer than ten amino acids were also discarded. Subsequently, complexes with covalently bound ligands as well as invalid ligand structures were filtered out. This reduced database of protein-ligand complexes is hand-curated: the primary citation associated with each structure is screened for binding affinity data while some “suspect ligands” were flagged for visual inspection, resulting in the final database of 5331 protein-ligand complexes.

The Binding MOAD has been expanded annually over the years by adding new protein-ligand complexes deposited on the PDB (together with binding affinity data), resulting in 23 269 total entries and 8156 entries with associated binding affinities in 2015 (Ahmed et al., 2014). In 2019, the Binding MOAD contained 32 747 structures comprising 9117 unique protein families and 16 044 unique ligands.

The Binding MOAD and the PDBbind databases are curated in a similar fashion, to the point that the two data sets could be compared to find and fix disagreements in overlapping systems (Hu et al., 2005). However, the Binding MOAD includes complexes with only binding cofactors, complexes with both a ligand and a cofactor present, and also includes high-quality complexes without binding affinity data (Hu et al., 2005).

Given that the development of the PDBbind was mostly driven by the development of scoring functions (Cheng et al., 2009; Li et al., 2014b,a; Su et al., 2018) while the development of the Binding MOAD was primarily driven by research on protein binding site prediction (Clark et al., 2020) and protein flexibility (Clark et al., 2019), it is more common to encounter the former in the development of ML and DL SFs. However, Binding MOAD can be certainly used for assessing the performance of scoring functions in binding affinity prediction (Xavier et al., 2016) and has been used to build the CSAR dataset discussed below.

### 3.2.1 CSAR

The CSAR dataset is a data set associated with the Community Structure-Activity Resource (CSAR) which has the goal of collecting high-quality data from both academia and industry to improve docking scoring functions and to organize community-wide assessments of current methods (Dunbar et al., 2011).

The first CSAR data set consisted of protein-ligand complexes from the PDB for which experimental binding affinities (*K*_*i*_ or *K*_*d*_ values) were available in the Binding MOAD database, augmented with data from the PDBbind; Dunbar et al. (2011) describe the CSAR data set as “the best of the PDB […] augmented with binding data from Binding MOAD and PDBbind”. The data set consists of 343 protein-ligand complexes which span binding affinities over several orders of magnitude.

The CSAR data set is subdivided into two subsets: Set 1, and Set 2. Initially, 2916 protein-ligand complexes were identified in the Binding MOAD database (version 2006) and filtered down to 1241 entries according to the quality of the crystal structures. Further processing consisted of the removal of ligands for which hybridization states and bond orders could not be automatically inferred, and for which the experimental binding affinity was expressed in terms of IC_50_ values. This resulted in a total of 309 complexes with associated *K*_*a*_, *K*_*d*_ and *K*_*i*_ values. Later on, an additional 1228 complexes from Binding MOAD (versions 2007 and 2008) were processed to obtain an additional 230 complexes with associated binding affinity data. After moving some complexes between the two groups to balance physicochemical properties, the final data set representing the initial release consisted of Set 1 (242 entries) and Set 2 (297 entries). Following community feedback, a more stringent quality assessment of the crystal structures was applied, thus reducing the size of the two sets, and errors concerning binding affinities were corrected. Following the CSAR benchmark exercise (Smith et al., 2011), the two sets were further processed resulting in the CSAR-NCS HiQ data set (September 2010), subdivided into Set 1 (176 entries) and Set 2 (167 entries). The CSAR-NCS HiQ data set consists of 52 protein targets with 2 or more structures and 191 targets with a single structure.

The CSAR-NCS HiQ dataset was subsequently updated with an additional 123 structures (set 3) applying the same criteria of the CSAR-NCS HiQ data set to structures in Binding MOAD added between 1/1/2009 and 12/31/2011.

The CSAR-NCS HiQ data set Dunbar et al. (2011), its update, and other data sets associated with the CSAR benchmark exercises (Damm-Ganamet et al., 2013; Dunbar et al., 2013; Smith et al., 2015; Carlson et al., 2016) can be downloaded from csardock.org or bindingmoad.org.

### 3.3 Astex Diverse Set

The Astex Diverse Set is another common data set encountered in the validation of protein-ligand scoring functions (Hartshorn et al., 2007), alongside the CASF and CSAR benchmarks. This data set contains 85 protein-ligand complexes, most of which are associated with experimentally measured binding potency.

The diverse set was obtained as follows. First, proteins from the PDB database were clustered based on sequence similarity leading to 9188 clusters of distinct proteins. Then, ligands bound to the clustered proteins were then screened to select high-quality structures of pharmaceutical or agrochemical interest and were filtered according to drug-likeliness criteria. The selected protein-ligand complexes were further assessed in terms of ligand clashes with the binding site residues, possible problems related to spurious interactions, and quality of the ligand electron density. This automated filtering procedure resulted in 427 clusters with high-quality protein-ligand complexes.

The final Astex Diverse Set was manually curated from the 427 clusters resulting in 85 complexes. Potency data for 74 of the 85 complexes was finally extracted from the literature.

### 3.4 Other Data Sets

The data sets described above are curated collections of binding affinities and structures and are therefore useful for the development and assessment of structure-based SFs for protein-ligand binding affinity predictions, both using classical and ML/DL scoring functions. However, there are several other data sets that might be useful to build and assess scoring functions, and some are briefly described below.

ChEMBL (Gaulton et al., 2011; Bento et al., 2013; Mendez et al., 2018) is a manually curated database of bioactive molecules, where data about drug-like molecules are collected together with results from bioactivity assays and genomic information. ChEMBL version 29 (10.6019/CHEMBL.database.29) contains data about 21 05 464 compounds and 14 554 targets. While ChEMBL is an extremely valuable resource and provides a large amount of binding affinity data, it does not contain structural data and it is, therefore, more commonly encountered in the development and assessment of ligand-based models (such as in Riniker and Landrum (2013a)).

The bioactivity data in ChEMBL is also exchanged with PubChem Bioassay (Wang et al., 2009, 2011) and BindingDB (Chen et al., 2001; Liu et al., 2007). The PubChem Bioassay database is a public repository containing bioactivity data for small molecules collecting more than 130 million assay results together with their protocols, while the BindingDB is a public database of experimental binding affinities between proteins (8,644 as of 8 November 2021) and drug-like molecules (1,023,385 as of 8 November 2021) which is accessible via a web interface. The BindingDB also contains 5988 protein-ligand crystal structures with associated binding affinity measurements.

Data sets released as part of the Drug Design Data Resources (D3R) Grand Challenges also constitute important datasets on which several ML and DL scoring functions have been designed or tested. D3R Grand Challenges promote the development and benchmarking of computational methods for binding pose and binding affinity prediction, by organizing blinded community challenges using high-quality data sets of pharmaceutical interest. The first D3R Grand Challenge was based on two targets (Gathiaka et al., 2016) using data from industrial drug discovery programs. Subsequent challenges (Gaieb et al., 2017, 2019; Parks et al., 2020) introduced novel targets and associated data for the blind prediction of binding poses, affinity rankings, and relative binding free energies. All the data sets are now easily accessible on the D3R website (drugdesigndata.org) as additional test sets for the development and evaluation of ML and DL scoring functions. Interestingly, in the D3R Grand Challenge 3 an increased number of ML methods was observed but overall they did not seem to perform any better than standard methods (Gaieb et al., 2019).

The databases that do not contain target structures are often employed to build ligand-based models or are used to put together new data sets with three-dimensional structures by generating different conformers for the ligand and collecting target structures from the PDB (Bernstein et al., 1977; Berman et al., 2000) to subsequently build structure-based models. For example, Boyles et al. (2021) recently released a new dataset—called the Updated DUD-E Diverse Subset—which combines data from the Directory of Useful Decoys Enhanced (DUD-E) data set (Mysinger et al., 2012) and ChEMBL.

Some data sets for binding affinity prediction discussed above are collected into benchmark data sets such as MoleculeNet (Wu et al., 2018) and Therapeutic Data Commons (Huang K. et al., 2021), which provide much-needed collections for the evaluation of different machine learning and deep learning methods for molecular properties prediction as well as drug discovery and development.

## 4 Machine Learning and Deep Learning Scoring Functions

**FIGURE 1:**
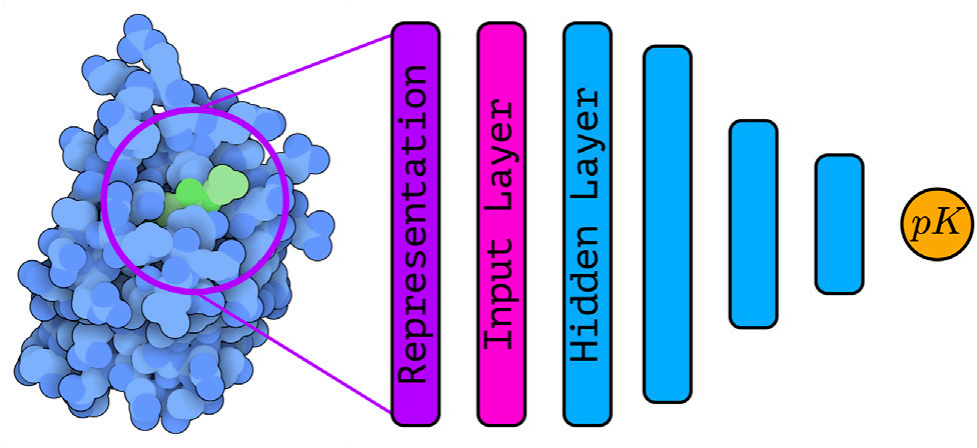
| Schematic representation of a structure-based deep learning architecture—with several hidden layers—for binding affinity prediction. The protein-ligand complex (PDB ID: 5S9H, rendered with Illustrate (Goodsell et al., 2019a)) is encoded into a suitable representation that is used as input to the deep learning architecture. Using a series of stacked hidden layers, a prediction of the binding affinity is finally obtained. The exact nature of the input layer as well as the hidden layers depends on the type of architecture under consideration.

Machine learning (or descriptor-based) scoring functions have been developed and used for decades (Brown 2020). The simplest “scoring functions”—more commonly known as QSAR (Quantitative Structure-activity Relationship) models—were based on a small set of handcrafted descriptors and simple models (such as multiple linear regression Morris et al. (1998); Böhm (1992)), and typically ligand-based. Later, other machine learning (ML) algorithms—such as support vector machines (SVM) (Boser et al., 1992; Cortes and Vapnik 1995), random forests (RFs) (Ho 1995, Ho 1998; Breiman 2001), and gradient boosting (Mason et al., 1999; Friedman 2002)—have been applied in attempt to learn non-linear relationships between descriptors and the binding affinity. Figure 1 shows a schematic representation of a structure based deep learning architecture for binding affinity prediction.

For an in-depth and rigorous introduction to the deep learning (DL) architectures described below the reader should consult Goodfellow et al. (2016), while classical ML methods are described thoroughly in Bishop (2006), to which we refer the interested reader. For a more hands-on introduction to both ML and DL the reader should consult Géron (2019).

### 4.1 Descriptors

Descriptors for ML and DL SFs can encode information about the ligand, about the protein, or about intermolecular interactions in the protein-ligand complex. Ligand descriptors are commonly used in cheminformatics applications, quantitative structure-activity relationship (QSAR) modelling, and ligand-based virtual screening (Lo et al., 2018). Ligand-based descriptors can be combined with descriptors for the protein commonly employed in ML-based protein engineering (Xu et al., 2020) to obtain a SF that combines separate information about the ligand and the protein. However, here we focus on structure-based descriptors that encode the protein-ligand complex as a whole and form the basis for structure-based SFs. Methods that combine separate ligand and protein descriptors (van Westen et al., 2011), known as “pair methods” or “proteochemometric models”, have been reviewed by Qiu et al. (2016) and more recently by Kimber et al. (2021).

One common distinction between ML and DL models is that the latter are usually based on a simpler representation and learn descriptors directly from the data; this distinction is however somewhat arbitrary and most DL models still require some pre-processing to convert atom types and coordinates in the correct format for the architecture being used. Here we briefly review structure-based descriptors commonly employed with ML algorithms as well as the input representation used in DL architectures.

One common type of descriptor employed with ML models is an interaction fingerprint (IFP). Structural interaction FPs (SIFts) encode the 3D structure of a protein-ligand complex into a one-dimensional binary vector (Deng et al., 2003). Seven different interaction types involving the ligand and binding site residues are identified and a FP for the whole protein-ligand complex is obtained by concatenating the binding bit string of each binding site residue. Simple ligand-receptor interaction descriptors (SILIRID) are instead obtained from binary IFPs by summing the bits corresponding to the same amino-acids (Chupakhin et al., 2014), thus resulting in a FP with 168 elements (20 amino acids and one cofactor, and 8 interaction types per amino acid). Da and Kireev (2014) developed structural protein-ligand interaction fingerprints (SPLIF), where protein-ligand atom pairs within 4.5 Å are identified and expanded into circular fragments described by extended connectivity fingerprints (ECFPs) (Rogers and Hahn 2010). In this way, protein-ligand interactions are encoded implicitly instead of needing explicit *ad-hoc* interaction classes and therefore can encode all local interactions (Da and Kireev 2014). Similarly, Wójcikowski et al. (2018) developed the protein-ligand extended connectivity (PLEC) FP which combines the ECFP environments of the protein and the ligand atoms in contact to describe protein-ligand interactions. The atomic features employed to construct PLEC FPs—atomic number, isotope, number of neighboring heavy atoms, number of hydrogens, formal charge, ring membership, and aromaticity—are the same used to construct ECFP, but only pairs of interacting atoms within 4.5 Å are considered. The FPs computed for ligand and protein atoms are hashed together to a final bit position (Wójcikowski et al., 2018). The PLEC FP implementation is available as part of the Open Drug Discovery Toolkit (Wójcikowski et al., 2015) and has been successfully used in combination with different ML models for binding affinity prediction (Wójcikowski et al., 2018). There are several other IFPs such as APIF (Pérez-Nueno et al., 2009), PADIF (Jasper et al., 2018), and PyLIF (Radifar et al., 2013).

Ballester et al. (2014) evaluated the impact of the choice of chemical descriptors on ML scoring functions. They showed that more complex descriptors do not necessarily lead to more accurate scoring functions and they identify and discuss the factors that might be contributing to this observation: modelling assumptions, co-dependence of representation and regression method, and data set features.

In structure-based methods, the goal is to exploit the 3D information of protein-ligand complexes. One natural representation of the 3D structure of protein-ligand complexes is the electron density, which is used in X-ray crystallography to model the structure of both the protein and the bound ligand. To encode information about the nuclei available from resolved structures, a representation that clearly encodes the spatial relationship between the protein and the ligand are three-dimensional (3D) grids which discretize volumetric data. The voxel occupancy is often defined as the sum (or maximum) of decaying density functions centered at the different atoms, while atoms of different types are represented in different grids—which can be thought of as a generalization of the RGB channels used in 2D images to represent the different colors. Different representations have been proposed, but they are mostly based on atom-centered density functions *g*(*r*; *t*_*i*_) centered at atom *i* of type *t* whose contributions are aggregated together:

$$G\left(\text{r};t,\text{R}\right)=\overset{N}{\underset{i}{⊕}}g\left(\|\text{r}-{\text{R}}_{i}\|;t_{i}\right)\delta_{t_{i},t}.$$ (6)

**r** represents the coordinates of the voxel, **R**_*i*_ represents the coordinates of atom *i*, while *δ*_*i*,*j*_ is Kronecker delta so that only atoms of type *t* contribute to *G*(**r**; *t*, **R**). *⊕* is an aggregation function such as sum or maximum.

In Jiménez et al. (2018),

$$g\left(r;t\right)=1-\exp\left[-{\left(\frac{R_{t}}{r}\right)}^{12}\right]$$ (7)

and the different channel represent hydrophobic, hydrogen-bond donor/acceptor, aromatic, ionizable, metallic, and excluded volume properties. *R*_*t*_ represents an atom type-dependent characteristic length, often set to the van der Waals radius. The properties are duplicated to represent protein and ligand atoms in different channels, and the density for different atoms in the same channel is aggregated by taking the maximum. In Ragoza et al. (2017a) and subsequent publications (Sunseri et al., 2018; Francoeur et al., 2020) the following functional form is used:

$$g\left(r;t\right)=\left\{\begin{array}{cc} e^{-2r^{2}/R_{t}^{2}}\;\; & 0\leq r<R_{t},\\ \frac{4}{e^{2}R_{t}^{2}}r^{2}-\frac{12}{e^{2}R_{t}}r+\frac{9}{e^{2}}\;\; & R_{t}\leq r<1.5R_{t},\\ 0\;\; & r\geq 1.5R_{t}, \end{array}\right.$$ (8)

and the different channels represent the different atom types from the Smina docking software (Koes et al., 2013), resulting in 16 channels for the receptor and 18 channels for the ligand (Ragoza et al., 2017a). Contributions from different atoms on the same channel are summed together.

The advantage of using 3D grid representations is that they encode clear spatial relationships between the different atom types and the computation can be performed very efficiently (Sunseri and Koes 2020) thus allowing on-the-fly data augmentation during training. However, grid representations have also several limitations. While computation of *G*(**r**; *t*) can be performed very efficiently, their dependence on the coordinate frame requires extensive data augmentation (Ragoza et al., 2017a) at increased computational costs, and the sparsity of some channels (such as the ones representing halogens or metals) implies wasteful computations. Additionally, the memory footprint of grid-based representations increases with the number of atom types. Despite the limitations, the close connection to the field of computer vision has led to the successful development of several SFs based on this representation, as discussed below.

For graph-based models such as graph neural networks (GNNs), descriptors are associated to atoms—the nodes of the graph—and bonds—the edges of the graph. A node descriptor is a vector containing information about the atom. An edge descriptor is a vector describing the chemical bond—such as the bond order. There are several descriptors employed in the literature, and they depend on the task at hand. For protein-ligand binding affinities, simple quantities related to an atom or a bond are commonly employed since higher-level features are learned by intermediate GNN layers (Feinberg et al., 2018). Such simple features for the nodes can include one-hot-encoded elements or atom types, formal charges, hybridization states, aromaticity, and other atomic properties (Jiang et al., 2021). Edge features can include both 2D and 3D information such as bond order, conjugation, bond length, and other bond properties (Jiang et al., 2021).

Descriptors commonly used in ML/DL for quantum chemistry have been successfully applied to the classification of active and decoys against different protein families (Bartók et al., 2017). Recently, the smooth overlap of atomic position (SOAP) descriptor (Bartók et al., 2013)—which allows comparing molecules across the structural and chemical space (De et al., 2016)—have been used together with Gaussian processes models to predict *p*IC_50_ values (McCorkindale et al., 2020). At the same time, atomic environment vectors developed for the ANI family of neural network potentials (Smith et al., 2017; Gao et al., 2020) and based on Behler-Parrinello symmetry functions (Behler and Parrinello 2007) have been used as descriptors of protein-ligand complexes for binding affinity predictions (Meli et al., 2021). Behler-Parrinello symmetry functions have also been employed as node features in GNNs for binding affinity prediction (Karlov et al., 2020) and inspired the atomic convolution architecture from Gomes et al. (2017). Both descriptors are strongly related (Musil et al., 2021) and provide a local descriptor of the structural and chemical environment of atoms in a way that is translationally and rotationally invariant.

Learned molecular representations also play an important role as descriptors (Chuang et al., 2020; Menke and Koch 2021). The characteristic of deep learning architecture is that useful and efficient internal representations are learned directly from the input data. Therefore, the fixed and *ad-hoc* descriptors or fingerprints described above can be substituted with learned representations. Yang et al. (2019) performed an extensive analysis of learned molecular representation for property predictions, showing that they achieve similar or better performance than fixed descriptors. While many learned representations for computational chemistry include only 2D information, learned representation for three-dimensional structures have been developed (Kuzminykh et al., 2018) but their application in structure-based drug discovery is still under-explored. The interest in DL architectures is that they can leverage the simple inputs described above (such as 3D atomic densities or coordinates and atom types) to automatically learn internally complex representations that can be used for molecular property prediction.

Some authors extracted descriptors from molecular dynamics (MD) trajectories, instead of using the crystal structure or docked poses, although the use of trajectory data remains rare (Wang and Riniker 2020). Yakovenko and Jones (2017) use atomic densities but trained their model on both docked poses and MD trajectory frames to obtain learned representations later used to predict LIE. Berishvili et al. (2019) developed 1D descriptors based on GROMACS (Berendsen et al., 1995; Abraham et al., 2015), AutoDock Vina (Trott and Olson 2009), and smina (Koes et al., 2013) terms to describe frames from MD trajectories. The descriptor for each frame where stacked together into a matrix of size *n*_descriptor_ × *n*_frames_, representing the whole MD trajectory.

A more in-depth overview of featurization strategies for protein-ligand interactions that are commonly employed in the development of ML and DL SFs is given by Xiong et al. (2021), while an overview of common molecular representations used in AI-driven drug discovery is provided by David et al. (2020).

### 4.2 Overview of Classical Machine Learning Scoring Functions

Classical ML algorithms such as SVMs and RFs have been used in quantitative structure-activity relationship (QSAR) modelling and in the development of structure-based scoring functions for a while (Ain et al., 2015; Muratov et al., 2020).

One of the earliest ML SFs for binding affinity predictions has been developed by Deng et al. (2004). The model combines protein-ligand atom pair occurrence and distance-dependent atom pair features with a kernel partial least squares method (K-PLS) (Rännar et al., 1994, Rännar et al., 1995) to predict *pK*_*d*_, demonstrating that structure-based descriptors combined with ML regression can be effective for protein-ligand binding affinity prediction on different complexes. Das et al. (2010) introduced property-encoded shape distribution signatures—descriptors encoding molecular shapes and property distributions on protein and ligand surfaces—which were used in combination with SVM to build a regression model. SVM-based regression was also used by Li et al. (2011) to develop two SFs, one based on knowledge-based potentials (SVR-KB) and another based on physicochemical properties of the protein-ligand complex (SVR-EP). Both SFs show very good performance on the CASF benchmark when compared to classical SFs.

RF models have been quite successful in the development of structure-based ML SFs. Ballester and Mitchell (2010) introduced a novel SF based on RF, called RF-Score. Protein-ligand complexes are described by a 36-dimensional feature vector storing the occurrence count of different protein-ligand atom pairs within a cutoff of 12 Å. The feature vector is used as input of a RF regression model predicting the binding affinity. Thanks to the use of the PDBbind benchmark (Cheng et al., 2009), RF-Score could be easily compared to 16 other SFs, showing that RF-Score is a very competitive scoring function. SFCScore^RF^ improved the performance of RF-Score by using a different and larger feature vector including ligand-based features (such as the number of rotatable bonds) and interaction-specific descriptors (Zilian and Sotriffer 2013).

Gradient boosting (Mason et al., 1999; Friedman 2002)—often combined with decision trees—is another popular ML technique used in the development of SFs, also thanks to the availability of high-quality open-source implementations such as XGBoost (Chen and Guestrin 2016) and LightGBM (Ke et al., 2017). Notable scoring functions based on gradient boosting are XGB-Score (Li et al., 2019b), AGL-Score (Nguyen and Wei 2019), and OPRC-GBT (Wee and Xia 2021). Shen et al. (2021) recently developed several XGBoost-based classifiers to assess the impact of cross-docked poses on the performance on pose-prediction, highlighting the importance of cross-docked poses for training of ML SFs with a broad applicability domain and increased robustness for pose-prediction.

Instead of learning the experimental protein-ligand binding affinity directly, Wang and Zhang (2016), used a RF model learning to correct the AutoDock Vina scoring function (Trott and Olson 2009), which represent a reasonable baseline—especially for docking and virtual screening. The resulting scoring function, called Δ_Vina_RF, retains the very good scoring performance of other ML SFs on scoring and ranking while also working well for docking and virtual screening. Δ_Vina_RF showed great performance in the CASF-2016 benchmark (with a Pearson’s *r* of 0.82 in the scoring task), but this superior performance can be partially attributed to the overlap between the training set and the CASF-2016 test set (Su et al., 2018).

ML-based scoring functions are still under active development both in terms of methodology and training data. For example, Boyles et al. (2019) showed that including ligand features obtained with RDKit into structure-based ML scoring functions consistently improves the performance in protein-ligand binding affinity prediction. Combining features from RF-Score (v3) with RDKit molecular descriptors improves Pearson’s correlation for the CASF-2016 scoring benchmark from 0.79 to 0.84 (Boyles et al., 2019). Another example of recently developed scoring function using classical machine learning regression models for binding affinity prediction is RASPD+ (Holderbach et al., 2020).

Several other classical machine learning algorithms such as kernel ridge regression, Gaussian processes (Williams and Rasmussen 1996; Rasmussen 2003), and other methods have been used in the development of structure-based scoring functions but they are not the focus of this review. The interested reader can consult Ain et al. (2015) and (Li H. et al., 2020) for a more in-depth review of machine learning scoring functions.

### 4.3 Feed-Forward Neural Networks

Feed-forward neural networks (also known as multilayer perceptrons (MLPs), fully-connected neural networks, artificial neural networks (ANNs), or simply neural networks (NNs)) consist in a series of linear layers combined with point-wise non-linearities called activation functions (Bishop 2006). Originally, feed-forward neural networks were inspired by the way neurons in the brain work (McCulloch and Pitts 1943; Widrow and Hoff 1960; Rosenblatt 1962).

The basic unit of a neural network is a “neuron” (perceptron, or node) and the neurons in a neural network are clustered in different layers that are stacked. The neuron *j* in layer *k* takes an input vector **x** ∈ **R** ^*N*^ returns an output

$$z_{j}^{\left(k\right)}=g\left(\overset{N}{\underset{i}{\sum }}w_{ji}^{\left(k\right)}x_{i}+b_{j}^{\left(k\right)}\right),$$ (9)

where $w_{ji}^{\left(k\right)}$ (weights) and $b_{j}^{\left(k\right)}$ (biases) for neuron *j* in layer *k* are learnable parameters to be determined during training and where *g*(⋅) is a non-linear function, called activation function. Neural networks are very expressive and can be regarded as universal approximators (Hornik et al., 1989), provided a large enough number of hidden neurons and some classes of activation functions (Bishop 2006).

Initially, neural networks were composed only of a small number of neurons with a single (hidden) layer between the input layer and the output layer but thanks to the development of algorithms able to train neural networks with multiple layers in a simple and efficient way (Rumelhart et al., 1986) neural networks became deeper and deeper (now called deep neural networks, DNNs) by staking together multiple hidden layers.

The use of simple and deep NNs for the determination of quantitative structure-activity relationships (QSAR) is not new (Salt et al., 1992; Dahl et al., 2014; Ma et al., 2015). One of the first use of NNs in binding affinity prediction was published by Artemenko (2008), where a subset of physicochemical descriptors and quasi-fragmental descriptors—describing pairwise statistics of interatomic distances—were selected using multiple linear regression and used as input of a feed-forward NN. NNs have been also successfully used for classification of actives and decoys. Durrant and McCammon (2010) introduced a NN-based SF—NNScore—to distinguish between well and poorly docked ligands as well as actives from decoys. NNScore was later extended to regression of binding affinities in NNScore 2.0 (Durrant and McCammon 2011b) thus providing a direct estimation of *pK*_*d*_. NNScore 2.0 uses terms from the Vina scoring function (Trott and Olson 2009)—to encode steric, hydrophobic, and hydrogen-bonding interactions–as well as BINANA features (Durrant and McCammon 2011a) as input and returns a estimate of *pK*_*d*_ as output.

Ashtawy and Mahapatra (2015) used a collection of NNs whose predictions are combined with the bagging (Breiman 1996)—bootstrap aggregation—or boosting (Freund and Schapire 1997; Friedman 2002) ensemble methods. The input features were obtained as a combination of classical scoring functions terms, together with features from RF-Score. Their BgN-Score and BsN-Score SFs perform significantly better on the PDBbind core set 2007 than classical SF and surpass SFs based on RFs.

Wójcikowski et al. (2018) showed that a MLP combined with their PLEC fingerprint can achieve very good performance on the CASF-2016 benchmark. However, they also show that the PLEC FPs perform equally well when using a simpler linear model instead of a neural network, confirming that well-crafted descriptors can be extremely powerful.

More recently, Zhu et al. (2020) developed a model for *pK*_*d*_ prediction where pairwise contributions are computed with a fully connected NN. Trained on the PDBbind 2018, they achieve a Pearson’s correlation coefficient of 0.75 and a RMSE of 1.44 on the CASF-2016 benchmark but the authors point out that there is a significant overlap between the test and training sets which might be boosting the performance of their model. Meli et al. (2021) used a collection of MLPs combined with a local representation of the atomic environment to predict protein-ligand binding affinities, reaching good performance on the CASF-2016 benchmark.

### 4.4 Convolutional Neural Networks

Convolutional neural networks (Fukushima 1980; Le Cun et al., 1989; Lecun et al., 1998; Krizhevsky et al., 2017) are a class of neural networks that tries to overcome some of the limitations of feed-forward neural networks, by using convolution operations instead of matrix multiplication in some of their layers (Goodfellow et al., 2016). Feed-forward neural networks use a one-dimensional vector as input which prevents the encoding of spatial relationships, and uses many parameters. CNNs are based on three main concepts (Bishop 2006): local receptive fields (inspired by the structure of the visual cortex (Hubel 1959; Hubel and Wiesel 1959)), weight sharing, and subsampling.

Local receptive fields are implemented in convolutional layers, where neurons in a layer do not receive the output of all neurons in the previous layer (as in fully-connected NNs) but only the ones in their local receptive field (Géron 2019). For two-dimensional grid-based inputs (such as images), the output of neuron at location (*i*, *j*) of feature map *k* of the convolutional layer *l* is given by (Géron 2019)

$$z_{i,j,k}^{\left(l\right)}=b_{k}^{\left(l\right)}+\overset{f_{h}^{\left(l\right)}}{\underset{u=1}{\sum }}\overset{f_{w}^{\left(l\right)}}{\underset{v=1}{\sum }}\overset{f_{n}^{\left(l-1\right)}}{\underset{k^{\left(l-1\right)}=1}{\sum }}x_{i^{\left(l-1\right)},j^{\left(l-1\right)},k^{\left(l-1\right)}}\cdot w_{u,v,k^{\left(l-1\right)},k}^{\left(l\right)},$$ (10)

with

$$\left\{\begin{array}{c} i^{\left(l-1\right)}=us_{h}^{\left(l\right)}+f_{h}^{\left(l\right)}-1,\;\\ j^{\left(l-1\right)}=us_{w}^{\left(l\right)}+f_{w}^{\left(l\right)}-1.\; \end{array}\right.$$ (11)

*f*_*h*_ and *f*_*w*_ are the height and the width of the receptive field (i.e. the size of the 2D convolutional kernel) while *s*_*h*_ and *s*_*w*_ represent the strides (i.e. the size of the displacement of the receptive field). $f_{n}^{\left(l-1\right)}$ denotes the number of feature maps in the previous layer (*l* − 1). $b_{k}^{l}$ is a bias term associated to feature map *k* while $w_{u,v,k^{\prime },k}^{\left(l\right)}$ denotes the weight term associated to the connection between the input located at (*u*, *v*) in feature map *k* ^(*l*−1)^ (relative to the neuron’s receptive field) and the neuron in feature map *k* of layer *l*. Both $b_{k}^{l}$ and $w_{u,v,k^{\prime },k}^{\left(l\right)}$ are learnable parameters to be determined during training. For clear depictions of the main building blocks of 2D CNNs we refer the reader to Dumoulin and Visin (2016).

Parameter sharing in a convolutional network comes from the fact that each weight $w_{u,v,k^{\prime },k}^{\left(l\right)}$ of the kernel is used at every position of the input, avoiding the need to learn a parameter for each input element as it is the case in MLPs. Parameter sharing does not reduce the computational complexity of the forward pass, but significantly reduces the number of parameters in the network (when the size of the convolutional kernel is much smaller than the size of the input) and therefore the associated memory footprint (Goodfellow et al., 2016).

Pooling layers—such as maximum pooling (Zhou et al., 1988), and average pooling—are often inserted after (activated) convolutional layers to make the representation approximately invariant to small translations (Goodfellow et al., 2016). Additionally, they reduce the size of the input of the next layer thus increasing the computational efficiency of the CNN, and are essential for dealing with inputs of varying size (Goodfellow et al., 2016).

Convolutional neural networks have been very successfully applied to different tasks in computer vision such as image classification (Krizhevsky et al., 2017) in the ImageNet challenge (Deng et al., 2009; Russakovsky et al., 2015).

Wallach et al. (2015) introduced a structure-based deep convolutional network for bioactivity prediction (classification into two activity classes) of small drug-like molecules against a target of interest. In their architecture, denoted AtomNet, the protein-ligand binding site was converted into a 3D grid (20 Å per side at 1 Å resolution) containing values related to structural features such as the number of atom types or protein-ligand descriptors such as SPLIF (Da and Kireev 2014), SIFt (Deng et al., 2003), or APIF (Pérez-Nueno et al., 2009). They showed improved performance in the ROC-AUC compared to their baseline, provided by the Smina docking software (Koes et al., 2013). Ragoza et al. (2017a) introduced a similar approach to distinguish good (low RMSD) from bad (high RMSD) docking poses using CNNs based on an atomic density representation of the binding site (see Eq. 8). This approach was later extended to include binding affinity predictions in a multitask learning framework (Sunseri et al., 2018)—both the binding affinity and the pose quality are predicted at the same time—and it was shown to provide a good correlation between experimental and predicted binding affinities for the CASF-2016 benchmark (Francoeur et al., 2020). The various pre-trained CNN scoring functions are integrated and readily available in the Gnina docking software (McNutt et al., 2021). Jiménez et al. (2018) took a similar approach—with a slightly different density representation, first introduced in DeepSite Jiménez et al. (2017)—for binding affinity prediction with their K_deep_ architecture and they achieved a very good correlation and low RMSE on the CASF-2016 benchmark. Interestingly, they analyzed the accuracy separately for the 58 different target classes of the CASF-2016 benchmark, revealing that accuracy is very sensitive to the specific protein used. Indeed, protein family-specific CNN models have been developed for virtual screening using a transfer-learning approach (Imrie et al., 2018).

Many other architectures for binding affinity predictions based on CNNs have been developed in recent years. Notable examples are Pafnucy (Stepniewska-Dziubinska et al., 2018), DeepAtom (Li Y. et al., 2019), OnionNet (Zheng et al., 2019; Wang S. et al., 2021) and OnionNet-2 (Wang Z. et al., 2021), and RoseNet (Hassan-Harrirou et al., 2020).

Pafnucy discretizes the binding site on a three-dimensional grid of 20 Å in side at 1 Å resolution and employs a set of 19 features including one-hot encoding of atom types (including selenium, halogens, and metals), hybridization state, number of bonds with heavy atoms, number of bonds with heteroatoms and a flag distinguishing protein and ligand atoms. DeepAtom uses a grid of 1 Å resolution to voxelize the binding site, with the same density representation of Jiménez et al. (2018) and using Arpeggio atom types (Jubb et al., 2017), but the architecture is inspired from ShuffleNet V2 (Ma et al., 2018). OnionNet (Zheng et al., 2019) also uses a deep convolutional neural network but the input features are based on intermolecular element-pair-specific contacts between ligand and protein atoms, which are grouped in different distance shells. Each shell is described by 64 features representing the intermolecular interactions—within the shell boundaries—between the protein and ligand for eight atoms types considered, and a total of 60 shells (of thickness 0.5 Å) is employed (Zheng et al., 2019). This idea was later extended in OnionNet-2 (Wang Z. et al., 2021), which uses protein residues types instead of protein atom types (increasing the number of features from 64 to 8 × 21 = 168). RoseNet (Hassan-Harrirou et al., 2020) uses an ensemble of CNNs—based on the ResNet architecture (He et al., 2016)—combining molecular mechanics energies from the Rosetta force field (Alford et al., 2017) voxelized onto a 3D grid (25 Å each side, at 1 Å resolution) and molecular descriptors—using an approach similar to *K*_deep_ with descriptors from AutoDock Vina (Trott and Olson 2009)—to predict absolute binding affinities.

CNNs can also be employed with lower-dimensional descriptors. For example, TopologyNet (Cang and Wei 2017) encodes the three-dimensional protein-ligand complex structure into one-dimensional element-specific fingerprints based on topological invariants. Such element-specific topological fingerprints, stacked together over multiple channels—like a one-dimensional image representation—are then used as input of a CNN, and achieve good performance on the CASF-2016 benchmark. The work was later extended to explore additional algebraic topology approaches (Cang et al., 2018).

CNNs have also been successfully applied to the related task of pose prediction. The CNN developed by Ragoza et al. (2017a) has been developed initially for pose prediction, and it was extended to binding affinity prediction on a later stage (Francoeur et al., 2020). Other notable examples are DeepBSP (Bao et al., 2021), which uses a 3D voxel representation of protein-ligand complexes to predict the RMSD between a docked ligand and its native pose—an idea previously explored by Aggarwal and Koes (2020)—and MedusaNet (Jiang H. et al., 2020), which uses CNNs to predict if a pose generated by docking is a good pose to stop the docking process earlier when *k* good poses are found thus reducing computational costs.

The application of CNNs in the prediction of protein-ligand binding affinities has been quite successful, as demonstrated by the methods discussed above. However, while CNNs are translational invariant they are not rotationally invariant and therefore require extensive data augmentation where the protein-ligand complex is randomly rotated before computing its associated grid representation. Data augmentation with CNNs has proven to be essential to prevent overfitting in pose prediction (Ragoza et al., 2017a), and the average over multiple random rotations can be used during inference thus reducing the variance of the predictions (Jiménez et al., 2018). Many concepts from geometric deep learning (Atz et al., 2021; Bronstein et al., 2021), such as CNNs that are equivariant to rigid body motions (Weiler et al., 2018), will spill more and more into the field of protein-ligand binding affinity prediction as well as virtual screening to overcome some of the limitations of standard CNNs by encoding relevant symmetries directly into the model.

### 4.5 Graph Neural Networks

Graph neural networks (GNNs) are a collection of DL architectures to work with data that can be represented as a graph (Bronstein et al., 2021). The vast majority of GNNs falls under three categories (Bronstein et al., 2021): convolutional (Defferrard et al., 2016; Kipf and Welling 2016), attentional (Monti et al., 2017; Veličković et al., 2017; Zhang et al., 2018), and message-passing (Gilmer et al., 2017; Battaglia et al., 2018). A graph G(V,E) is composed of a set of vertices *v*_*i*_ ∈ *V* and a set of edges *e*_*ij*_ ∈ *E* connecting the vertices. Features **x** are associated to vertices (and, optionally, edges) and such features are subsequently updated as follows:

$${\text{h}}_{u}=\phi \left({\text{x}}_{u},\underset{v\in N_{u}}{⊕}\psi \left({\text{x}}_{u},{\text{x}}_{v}\right)\right)$$ (12)

where *ϕ* and *ψ* are learnable functions (often learnable affine transformations with activation functions (Bronstein et al., 2021)) and where *⊕* represents a permutation-invariant function allowing the aggregation of features (such as sum, mean, and maximum (Bronstein et al., 2021)) over the neighborhood $N_{u}$ of node *u*. *ψ* is a message-passing function (which can be generalized to include edge features as well), while *ϕ* is a vertex update function. It is possible to learn edge features as well by introducing a hidden representation **h**_*uv*_ for the edges (Kearnes et al., 2016; Gilmer et al., 2017).

Since molecules can be naturally represented as graphs—with nodes in the graphs representing different atoms and edges in the graph representing the chemical bonds between such atoms—GNNs are well suited to be applied in the field of chemistry (Atz et al., 2021). Message-passing GNNs, which are the most general flavor, have been successfully applied in quantum chemistry applications (Schütt et al., 2018; Qiao et al., 2020; Schäfer et al., 2020; Christensen et al., 2021). GNNs have also been applied to several molecular property predictions (Gaudelet et al., 2021), including bioactivity and protein-ligand binding affinity.

Gomes et al. (2017), inspired by the work of Behler and Parrinello (2007), developed an atom type convolution that uses a neighbor-listed distance matrix to automatically extract features about local chemical environments and combine this information with radial pooling to downsample the output of the atom type convolution. Essentially, the atom type convolution performs a graph convolution on the nearest neighbors graph in three-dimensional space. The resulting features are then passed to a collection of fully connected layers (all with the same weights and biases) to predict atomic contributions to the energy, which are summed together to obtain the total Gibbs free energy. To predict the binding free energy, three weight-sharing networks are used (one each for *G*_complex_, *G*_protein_ and *G*_ligand_) and the results are then combined as

$$\Delta G_{\text{complex}}=G_{\text{complex}}-G_{\text{protein}}-G_{\text{ligand}}$$ (13)

so that the whole architecture directly incorporates the thermodynamic cycle.

In PotentialNet (Feinberg et al., 2018) the node updates are of the form

$$h_{u}^{\left(k\right)}=\text{GRU}\left(h_{u}^{\left(k-1\right)},\overset{N_{\text{et}}}{\underset{e}{\sum }}\underset{v\in N^{e}\left(\nu_{i}\right)}{\sum }{\text{NN}}^{e}\left(h_{v}^{\left(k-1\right)}\right)\right)$$ (14)

where GRU is a gated recurrent unit (Hochreiter and Schmidhuber 1997; Cho et al., 2014; Chung et al., 2014), NN^*e*^ is a trainable NN for edge type *e*, and $N^{e}\left(\nu_{i}\right)$ denotes the neighbors of edge type *e* for atom *i*. Several updates are concatenated into different stages: in the first stage information is propagated only between nodes linked by a covalent bond, in the second stage information is propagated between non-covalent and covalent bonds and finally, everything is aggregated by a ligand-based graph gather. The first step essentially produces learned (bond-based) atom types, while the second step includes both bond and spatial information between the atoms (Feinberg et al., 2018). In stage three, all learned features for the ligand atoms are summed together and the resulting vector is used as input of a fully-connected neural network to produce the final prediction.

The graphDelta architecture uses a graph-based representation for the ligand and incorporates information about the target in the node features (Karlov et al., 2020). The node features represent radial and angular Behler-Parrinello atom centered symmetry functions (ACSFs) (Behler and Parrinello 2007), combined with a message-passing neural network. With enough training epochs, they achieve a Pearson’s correlation coefficient of 0.87 and a RMSE of 1.05 in the CASF-2016 benchmark for binding affinity prediction.

Li et al. (2021) developed a structure-aware interactive GNN which combines polar coordinate-inspired graph attention layers and pairwise interactive pooling. The graph attention layers leverage distances between nodes and angles between edges to iteratively update node and edge embeddings while preserving distance and angle information among atoms. The pairwise atomic type-aware pooling layer is then used to gather interactive edges to capture long-range interactions. Their model, called SIGN, achieves good results on the CASF-2016 benchmark for binding affinity prediction as well as the CSAR-NRC HiQ set.

Son and Kim (2021) developed GraphBAR, where a graph is constructed from all ligand atoms and protein atoms within 4 Å from the ligand (limited to a maximum of 200 nodes, with zero-padding of the adjacency matrix for smaller graphs). Node features consist of one-hot encoded atom types, atom hybridization states, number of neighboring atoms (heavy atoms and heteroatoms), and well as partial charges, stored in a 200, ×, 13 feature matrix. Multiple binary adjacency matrices are used to encode different interaction shells with fixed distance intervals. A graph convolution block is applied to each adjacency matrix together with the feature matrix pre-processed by a fully-connected layer. The outputs of the graph convolutional blocks are concatenated and a fully connected layer produces the final prediction. The model shows similar performance to Pafnucy (Stepniewska-Dziubinska et al., 2018), but the training time appears to be considerably shorter (Son and Kim 2021).

Jiang et al. (2021) developed InteractionGraphNet, where two independent graph convolution modules are stacked to sequentially learn intramolecular and intermolecular interactions using three molecular graphs (one for the ligand, one for the protein, and one for the protein-ligand complex). The protein-ligand bipartite graph is built using protein and ligand atoms within 8 Å of each other. At first, a series of message passing iterations is employed to update the node features in the protein and ligand graphs. Then, these learned node features are used as initial node features for the protein-ligand graph on which edge features representing non-covalent interactions are updated. The learned edge features on the protein-ligand graph, representing the non-covalent interactions between the protein and the ligand, are finally pooled together and used for downstream prediction tasks: binding affinity prediction, virtual screening and pose prediction. For binding affinity prediction, InteractionGraphNet shows good results on the CASF-2016 benchmark, although several systems were removed from the test set.

Moesser et al. (2022) recently developed a simple but effective way to include protein-ligand interactions into ligand-based graphs. Their protein-ligand interaction graphs (PLIGs) representation featurize an atom node in the molecular graph by including both atom properties and atom-atom contacts with protein atoms. Combined with the GAT architecture (Veličković et al., 2017), their model reaches a very good performance on the CASF-2016 benchmark.

Moon et al. (2022) used GNNs in a very interesting way. Instead of using standard and general architectures, Moon et al. (2022) included parametrized physics-based equations in the model architecture, to incorporate the appropriate inductive bias with the goal of improving model generalization by forcing the model to learn the underlying chemical interactions. A GNN is used to update node features across covalent bonds and intermolecular interactions, which are then used—together with pairwise distances—as input of physics-based parametrized equations describing intermolecular interactions as well as entropy loss. The parameters of the physics-informed equations are learned during training and contribute to model generalization.

GNNs have been also successfully applied for structure-based virtual screening (classification of actives and decoys) as well as pose prediction (classification of binding poses), as demonstrated by Lim et al. (2019), Morrone et al. (2020), and Stafford et al. (2022). The use of GNNs—and, more generally, geometric deep learning—in drug discovery and drug development is a very active area of research and a recent overview on several different applications beyond the narrow scope of this review is given by Gaudelet et al. (2021).

### 4.6 Other Methods

Above we briefly described widely used families of deep learning architectures—MLPs, CNNs, and GNNs—and their application on the development of structure-based scoring functions. One important omission is recurrent neural networks (RNNs) (Rumelhart et al., 1986; Hochreiter and Schmidhuber 1997; Graves 2012), which are suited to learn from sequential data (such as language or time series). RNNs are also applied to protein-ligand binding affinity prediction (Karimi et al., 2019) but they usually employ unrelated representation for the protein (often the sequence of amino acids) and the ligand (SMILES strings or related representations). As mentioned above, proteochemometric or pair models (Lenselink et al., 2017; Feng et al., 2018; Öztürk et al., 2018; Shin et al., 2019; Jiang M. et al., 2020; Nguyen et al., 2020; Yang et al., 2022) are outside the scope of this review and the reader can find more information in Kimber et al. (2021).

Similarly to proteochemometric models, which combine different—often learned—representations for the protein and the ligand, protein-ligand binding affinity predictions can also benefit from the use of complementary representations of the complex. Jones et al. (2021) combine learned representations of the protein-ligand complex obtained with CNNs and GNNs using mid-level or late deep fusion (Roitberg et al., 2019).

Seo et al. (2021) recently developed BAPA, an architecture based on 1D CNNs combined with an attention layer. The protein-ligand complex is encoded into a 1D descriptor of contacts between the protein and ligand atoms and processed using a 1D CNN to obtain learned features, which are then concatenated with terms from the AutoDock Vina scoring function. The learned features are then encoded into a latent representation using a MLP. The encoded vector is then passed to an attention layer. As described by Chen et al. (2018), an attention layer computes a weighted sum of input values, where the weights are determined based on the relevance of the different input components. In BAPA, the goal of the attention layer is to extract the components of the input important for binding affinity prediction in a context vector. The encoded and context vectors are then concatenated an used by an MLP to obtain the final prediction. Wang Y. et al. (2021) also used self-attention in their PointTransformer architecture. The use of the attention mechanism (Bahdanau et al., 2014; Luong et al., 2015) in binding affinity prediction is also found in proteochemometric models (Karimi et al., 2019; Zhao et al., 2019).

A totally different approach from the data-driven ones reviewed above is to use physics-based methods for the computation of binding free energies accelerated or improved using ML and DL. Thanks to the recent developments in ML force fields (Unke et al., 2021), accurate alchemical free energy calculations based on such force fields are starting to appear (Rufa et al., 2020; Wieder et al., 2021). ML-based corrections to conventional free energy calculations will also play an important role in reaching good prediction accuracy of protein-ligand binding free energies (Dong et al., 2021). While such methods are outside the scope of this review, we believe the exploration and development of ML and DL methods in the field of free energy calculations will provide very interesting outcomes in the coming years, by getting the methodology closer to chemical accuracy while significantly reducing computational costs.

## 5 Training and Evaluation

### 5.1 Back-Propagation, Regularization and Transfer Learning

Deep learning architectures for supervised learning are usually trained with gradient-based optimisation of a loss (or cost, or error) function that represents some measure of the prediction error (such as the mean squared difference between predicted and expected values). The weights and biases (trainable or learnable parameters) of the model are initialized from a random distribution or in a data-driven fashion (Narkhede et al., 2021), and they are iteratively adjusted by gradient-based optimisation techniques (such as stochastic gradient descent (Bottou et al., 1998)) to minimize a loss function.

Rumelhart et al. (1986) developed an algorithm called backpropagation, which allows computing the gradient of the loss function with respect to the parameters of the model (weights and biases) in an automated and efficient way. The algorithm consists of a forward pass computing the output of each component of the neural network, and the final output is used to evaluate the loss function. Then, the error is propagated backward using the chain rule of calculus to compute the gradients of the loss function with respect to each parameter of the network. The backpropagation algorithm is explained in detail in Goodfellow et al. (2016).

Modern deep learning frameworks such as PyTorch (Paszke et al., 2019; Li S. et al., 2020) and TensorFlow (Yu et al., 2018) usually require one to define only the forward pass, and gradients of the loss function can be easily and automatically computed with respect to any parameter. The availability of open-source, well-designed, and easy-to-use deep learning frameworks certainly contributed to the increased application of DL in different areas of research, including drug discovery.

Given the large number of parameters, DL architectures are often subject to the pitfalls of overfitting. To prevent overfitting, several techniques are commonly employed such as early stopping (Caruana et al., 2001), and the use of dropout layers (Srivastava et al., 2014).

Oftentimes, especially in the field of drug discovery, there is interest in models that are not completely generalizable but work well in specific cases such as specific protein families. Once a model has been trained on a general data set, it is possible to fine-tune the learned parameters to improve performance for specific tasks. Transfer learning (Bozinovski and Fulgosi 1976) methods can be subdivided in four classes (Pan and Yang 2010): instance-based, feature-based, parameter-based, and relation-based. Deep transfer learning, the combination of transfer learning and deep learning architectures (Tan et al., 2018), is commonly exploited in drug discovery applications where learned representations are employed in different tasks (feature-based transfer learning) or where pre-trained models are fine-tuned for specific tasks (parameter-based transfer learning). The latter technique has been used successfully to develop protein family-specific models for virtual screening (Imrie et al., 2018), for example. An overview of transfer learning in drug discovery is given by Cai et al. (2020).

Multitask learning, which is closely related to transfer learning, consists in learning multiple endpoints at the same time using a shared representation (Ramsundar et al., 2017). Multitask learning can be used for the development of ML and DL SFs for both pose prediction (docking) and binding affinity prediction (scoring) (Ashtawy and Mahapatra 2017; Francoeur et al., 2020).

### 5.2 Evaluation

The models for protein-ligand binding affinity prediction discussed above consist of regression models, which given a protein-ligand complex as input return a real-valued estimate of the binding affinity (usually *pK*_*d*_, *pK*_*i*_, or *pIC*_50_).

In the CASF benchmark, arguably one of the most used benchmarks for the development of scoring functions, the scoring power of a scoring function is measured in terms of correlation between experimental and predicted values. This correlation is measured quantitatively using Pearson’s correlation coefficient *r*, defined as

$$r=\frac{\sum_{i}\left(x_{i}-\bar{x}\right)\left(y_{i}-\bar{y}\right)}{\sqrt{\sum_{i}{\left(x_{i}-\bar{x}\right)}^{2}}\sqrt{\sum_{i}{\left(y_{i}-\bar{y}\right)}^{2}}},$$ (15)

where (*x*_*i*_, *y*_*i*_) are the predicted and experimental values of the binding affinity, while $\bar{x}$ and $\bar{y}$ are the corresponding averages on the whole data set. A Pearson’s *r* of 1.0 indicates perfect correlation, while a Pearson’s *r* of 0.0 indicates no correlation. The Pearson’s correlation coefficient is often accompanied by the root mean squared error

$$\text{RMSE}=\sqrt{\frac{1}{N}\overset{N}{\underset{i}{\sum }}{\left(x_{i}-y_{i}\right)}^{2}},$$ (16)

or the mean absolute error (MAE)

$$\text{MAE}=\frac{1}{N}\overset{N}{\underset{i}{\sum }}|x_{i}-y_{i}|,$$ (17)

where *N* is the total number of samples in the test set.

The predicted value of the protein-ligand binding affinity can also be used to rank compounds, usually against the same target. Common metrics to evaluate the ranking power of a scoring function are rank correlation coefficients such as Spearman’s *ρ* (Spearman 2010) and Kendall’s *τ* (Kendall 1938). The Spearman’s rank correlation coefficient is defined as (Spearman 2010)

$$\rho =\frac{\sum_{i}\left(r_{x_{i}}-\overset{–}{r_{x}}\right)\left(r_{y_{i}}-\overset{–}{r_{y}}\right)}{\sqrt{\sum_{i}{\left(r_{x_{i}}-\overset{–}{r_{x}}\right)}^{2}}\sqrt{\sum_{i}{\left(r_{y_{i}}-\overset{–}{r_{y}}\right)}^{2}}},$$ (18)

which is similar to Pearson’s *r* but uses the predicted and experimental ranks $\left(r_{x_{i}},r_{y_{i}}\right)$ —and the corresponding sample averages—instead of using directly the predicted and experimental values (*x*_*i*_, *y*_*i*_). The other difference is that the Pearson’s correlation coefficient is usually computed on the whole data set, while the Spearman’s rank correlation coefficient (and other rank correlation coefficients) are often disaggregated by target. This is the case for the CASF benchmarks, for example (Su et al., 2018). Another way to quantify the ranking power of a scoring function is the predictive index (PI) introduced by Pearlman and Charifson (2001) and defined as

$$\text{PI}=\frac{\sum_{i}\sum_{j>i}W_{ij}C_{ij}}{\sum_{i}\sum_{j>i}W_{ij}}$$ (19)

where *W*_*ij*_ = |*y*_*i*_ − *y*_*j*_| is the absolute difference between the experimental binding data of ligands *i* and *j* and where *C*_*ij*_ is defined as (Pearlman and Charifson 2001)

$$C_{ij}=\left\{\begin{array}{cc} 1\; & \;\text{if }\frac{y_{j}-y_{i}}{x_{j}-x_{i}}<0,\\ -1\; & \;\text{if }\frac{y_{j}-y_{i}}{x_{j}-x_{i}}>0,\\ 0\; & \;\text{if }x_{j}-x_{i}=0. \end{array}\right.$$ (20)

The weights *W*_*ij*_ reflect the fact that ranking incorrectly compounds with similar experimental binding affinities is less detrimental than ranking incorrectly compounds with vastly different binding affinities. As for Spearman’s and Kendall’s rank correlation coefficients, the PI is bound on the interval [ − 1, 1] (with 0 indicating random predictions).

Confidence intervals for the correlation coefficients described above can be computed using bootstrapping (Efron, 1992). For the CASF-2016 benchmark this is easily done with the provided analysis scripts (Su et al., 2018). The very important topics of calculation of confidence intervals and comparison of different models are discussed at length in Nicholls (2014) and Nicholls (2016) and while we are concerned with regression models in this review, we point the reader interested in the comparison of classification models to Patrick Walters (2021).

Given that the gradient-based training described above depends on the initialization of the parameters of the model, oftentimes multiple models are trained starting from different weights and using different seeds for the random number generator (used for random weight initialization, random shuffling of examples, …), and the final prediction consists on a combination of the results of the different models (often an average). This ensemble approach has been shown multiple times to improve predictions of machine learning and deep learning models (Hansen and Salamon 1990; Ashtawy and Mahapatra 2015; Ericksen et al., 2017; Francoeur et al., 2020; Kwon et al., 2020; Meli et al., 2021). More generally, a consensus score amongst multiple models (also with different architectures) can be used as well (Druchok et al., 2021), and the average between different models (different architectures and/or different training data sets) has been shown to improve pose predictions with CNN scoring functions (McNutt et al., 2021). While the average across different models is often used to estimate the performance of the ensemble, the standard deviation across predictions gives information about their stability and can be used as a diagnostic tool. Low standard deviations are expected within the domain of applicability of the models, while large standard deviations are often a symptom of poor generalizability.

Consensus scoring is not a new idea applicable only to machine learning and deep learning models; several flavors of consensus scoring have been successfully applied in combining different classical docking scoring functions for a long time (Charifson et al., 1999; Wang and Wang 2001; Clark et al., 2002). It is now commonly applied in ML and DL scoring functions to improve prediction performance.

Uncertainty quantification is an important field of machine learning and deep learning research and applications in drug discovery are a very active area of research. Some uncertainty quantification methods such as Monte Carlo dropout (Gal and Ghahramani 2016) remain under explored. Recently, evidential deep learning (Amini et al., 2020) has been applied to uncertainty quantification in DL-based QSAR (Soleimany et al., 2021). Soleimany et al. (2021) show that evidential deep learning allows to obtain predictions where uncertainty correlates with error and that uncertainty can be employed to perform sample-efficient training. Given the flexibility and scalability of the approach, which can be easily incorporated into existing architectures, this approach might contribute to the development of SFs in the near future.

### 5.3 Cross-Validation and Data Splitting

Very important aspects to consider when training and evaluating a new model are the size of the training set, the overlap between training and test sets, and the data set bias. These aspects need to be carefully evaluated, to properly assess the performance and generalizability of a new model.

The size of the training set affects the performance of ML and DL models and several authors noticed that including more examples in the training set—even of a lower quality, such as lower-resolution structures—improves model performance (Li et al., 2015; Francoeur et al., 2020). Learning curves, which show the prediction error as a function of the number of training examples, are commonly employed to evaluate and compare ML and DL methods in molecular properties prediction but they remain somewhat uncommon in the evaluation of structure-based models for binding affinity prediction, probably because of the much smaller size of the data sets available for training and evaluation.

The similarity between training and test sets has also a very high impact on the performance of structure-based models (Kramer and Gedeck 2010; Li and Yang 2017) and a careful model evaluation needs to take this similarity into account to avoid artificially inflated performance. Li and Yang (2017) studied the impact on ML SFs of protein structural and sequence similarity between the training and test. In their study, they remove training proteins that are highly similar to the ones in the test set, as evaluated by structural and sequence alignment. They concluded that ML SFs do not outperform classical scoring functions after removal of proteins from the training set with a high degree of similarity with the test set and therefore they attributed the higher performance of ML SFs compared to classical SFs to the existence of similarities between proteins in the training and test sets. Li et al. (2018), however, performed a similar study and concluded that the good scoring power of RF-Score is not exclusively due to a high number of similar proteins, although when sufficiently similar targets are present in both the training and test set ML scoring functions perform consistently better than classical scoring functions (Shen et al., 2020b). Additionally, ML scoring functions are able to exploit new data points as they become available, while classical scoring functions seem unable to exploit the large volumes of structural and interaction data available nowadays; incorporating a larger proportion of similar complexes to the training set does not seem to make classical SFs more accurate, according to Li et al. (2019b).

Boyles et al. (2019) and Su et al. (2020) both developed sub-sets of the PDBbind data set to carefully evaluate the effect of protein and ligand similarities on the performance of models trained on PDBbind and tested on the CASF data set. Boyles et al. (2019) evaluated ligand similarity using the Tanimoto similarity between Morgan fingerprints of each pair of ligands while protein similarity was evaluated with by sequence identity. Su et al. (2020) also used protein sequences to determine protein similarity, but used 3D shape similarity (Vainio et al., 2009) to evaluate the similarity between ligands. Additionally, Su et al. (2020) also evaluated binding site similarity—the binding site might be preserved, in contrast to the overall protein sequence—using structural descriptors including residue types and interatomic distances (Yeturu and Chandra 2008). Both groups confirmed the strong dependence on the similarity between the training and test set of the performance of ML scoring functions, which poses a challenge in the comparison of ML and DL SFs with classical SFs. While these considerations are very important in the development of new methods and it is important to take them into account when comparing different models, in practical applications the similarity between the training set and the system under investigation can be exploited to obtain superior predictions compared to classical SFs. For example, Li et al. (2021a) argue that the performance of ML scoring functions is underestimated due to the artificial removal of similarities between the training and tests sets and put forward a new benchmark with tries to mimic prospective binding affinity predictions. However, it is important to keep in mind that ML and DL SFs might be less effective when dealing with novel targets or small molecules (Su et al., 2020), and the applicability domain needs to be clearly defined.

Very recently, Ji et al. (2022) developed a free and open-source Python package that allows to curate dataset for benchmarking out-of-distribution (OOD) algorithms in the context of protein-ligand binding affinity predictions. The authors highlight a significant performance gap between in-distribution and OOD experiments, highlighting the need for new and domain-specific techniques allowing better OOD generalization.

Another way to elucidate the performance of ML and DL SFs in light of similarities and dissimilarities between the training test set is to use clustered cross-validation. *K*-fold cross-validation is an established technique for the evaluation of ML and DL models (Arlot and Celisse 2010) that consists of randomly splitting the training set into *K* different sets and use, in turn, *K* − 1 sets for training and the remaining set for validation/testing. Francoeur et al. (2020) evaluated the performance of their CNN scoring function using cross-validation with clusters based on protein sequence and ligand fingerprint similarities (for the models trained using PDBbind) and also concluded that evaluations based on the PDBbind core set are overly-optimistic and therefore a rather poor measure of the model’s ability to generalize to novel target and small molecules.

Finally, care should be taken in the presence of data set bias. One of the simplest forms of bias in current data sets is that published binding affinities tend to come from publications where potent binders were identified. Therefore, the distribution of binding affinities available for training might be skewed to potent binders and the trained model might be unable to predict binding affinities for weak binders. Bias can also be introduced in the construction of the training and test sets. For example, for the classification of actives and decoys on the DUD-E data set (Mysinger et al., 2012) it has been shown that analogue bias together with easily distinguishable decoys (decoys bias) result in CNN SFs exploiting only ligand information even when structure-based information is provided (Chen L. et al., 2019). Yang et al. (2020) also caution about the use of DUD-E to train ML and DL models to predict protein-ligand interactions but point out that the data set can still serve as an independent test set. Sieg et al. (2019) analyzed the problem of data set bias in-depth and proposed guidelines to recognize biases and develop robust models. Yang et al. (2020) suggest to evaluate the performance of ligand-only and protein-only models to better understand what ML and DL methods are learning from protein-ligand complexes.

The problem of unnoticed biases in the dataset that are exploited on learning by complex DL models is related to the infamous “black box” nature of some models.

## 6 Explainable AI

As mentioned in the previous section, the “black box” nature of some models poses serious challenges in the identification of biases in the data sets and often prevents a deeper understanding of the model predictions and especially of its failures. In recent years, a lot of research effort has been devoted to model interpretability and explainable artificial intelligence (XAI) (Lipton 2018; Gunning et al., 2019; Murdoch et al., 2019).

To unpack the predictions of CNN-based scoring functions, several authors focused on feature attribution methods. For example, Stepniewska-Dziubinska et al. (2018) estimated feature importance of the different input channels by looking at the weight distributions of the convolutional filters of the first layer. Hochuli et al. (2018) also looked at the weights of the convolutional filters of the first layer, which can give some insight on how the model uses the different input atom types. Hochuli et al. (2018) used additional established methods for feature attribution—such as gradient computation, a modified version of layer-wise relevance propagation (Bach et al., 2015), and masking (Štrumbelj et al., 2009; Szegedy et al., 2013)—combined with visualization of the protein-ligand complex, showing that each method provides some insight into their CNN scoring function.

Gradient-based feature attribution methods, which allow to determine (local) feature importance, consist in computing the gradient of the prediction with respect to the input. In DL models, such gradients are readily available thanks to the automatic differentiation machinery of modern deep learning frameworks. Interestingly, the gradients of the SF with respect to atomic coordinates can be used to perform ligand pose optimization in the context of docking (Ragoza et al., 2017b). Masking, a perturbation-based feature attribution approach, consists in removing part of the input in order to measure the change in output. Masking can be performed on single atoms or fragments and whole protein residues. While masking approaches are close to chemical intuition and directly estimate feature importance of different atoms or functional groups, they are computationally expensive since they require several evaluations per input.

Hochuli et al. (2018) show that feature attribution methods are able to identify important atoms in the ligand and this information can potentially be employed to optimize protein-ligand interactions during lead optimization. However, it is not always clear why particular atoms are highlighted as important (Hochuli et al., 2018). More recently, Varela-Rial et al. (2022) applied the integrated gradient feature attribution technique (Sundararajan et al., 2017) to their *K*_deep_ model, confirming that the model can generally learn meaningful interactions, but that in some cases important interactions where ignored or protein residues far from the ligand were highlighted. The fact that residues far from the ligand are highlighted as important suggest that in some cases the model is exploiting protein similarity instead of important physical interactions between the protein and the ligand.

The feature attribution methods shortly described above in the context of CNN SFs can be applied to other models as well. For example, gradient-based attribution has been applied in combination with GNNs to identify pharmacophoric features involved in ligand binding (McCloskey et al., 2019), while Cho et al. (2020) applied layer-wise relevance propagation to explain the predictions of their InteractionNet model.

For GNNs, there are several XAI methods specifically tailored for such architecture (Jiménez-Luna et al., 2020) and it is currently a vibrant area of research (Baldassarre and Azizpour 2019; Yuan et al., 2020; Agarwal et al., 2021). XAI methods for graphs can be classified in two categories (Jiménez-Luna et al., 2020): sub-graph identification, and attention-based (Veličković et al., 2017) approaches. Sub-graph identification is useful to identify a compact sub-graph structure as well as a small subset of node features that contribute strongly to the model prediction (Ying et al., 2019). While GNN-based XAI has seen several applications in the prediction of molecular properties and reactivity (Ryu et al., 2018; Coley et al., 2019; Preuer et al., 2019; Jiménez-Luna et al., 2021b), its consistent application to GNN-based structure-based scoring function is still under-explored.

Uncertainty quantification, briefly discussed above in the context of model evaluation, is also an important XAI technique with the goal of quantifying the reliability of a prediction. Ensemble approaches are currently employed in most applications but probabilistic approaches such as evidential deep learning (Amini et al., 2020; Soleimany et al., 2021) will play a major role in the future.

Model interpretability is also important for classical ML methods—such as RFs, and SVMs—and QSAR models (Riniker and Landrum 2013b; Marchese Robinson et al., 2017; Rodríguez-Pérez and Bajorath 2019; Sheridan 2019), that are not the focus of this review. Several XAI methods are model-agnostic and therefore work with several ML and DL methods. However, it is worth mentioning that the heavily pre-processed features—such as interaction fingerprints discussed above—often used in combination with classical ML methods might render the models less interpretable than complex DL methods (Lipton 2018).

XAI approaches have the potential to transform the application of DL in real drug discovery applications. Being able to explain why a particular prediction is relevant and interesting would facilitate the adaptation of computational models in experimental pipelines. However, several limitations of XAI remain. For example, XAI approaches are still under active development and research, and often the methods need to be carefully tailored to the problem at hand. Additionally, as pointed out by Jiménez-Luna et al. (2020), there is no method that combines all desirable features of XAI—transparency, justification, informativeness, and uncertainty estimation—and therefore current applications often rely on consensus approaches between methods possessing different desirable features.

A recent, extensive, and very accessible review of XAI applications in drug discovery is given by Jiménez-Luna et al. (2020), which also outline recent advances in the field of XAI that are yet to be applied to chemistry or drug discovery. However, the field is moving at a fast pace and some of the methods without any reported application in drug discovery in Jiménez-Luna et al. (2020)—such as instance-based methods—are now starting to be applied successfully (Wellawatte et al., 2022).

## 7 Discussion and Conclusion

In this review, we focused on structure-based scoring functions for binding affinity prediction based on deep learning, many of which have been developed in recent years. The large number of recently developed SFs (see Table 2 for a non-exhaustive list) is a testament to this rapid and fast-moving field. Li H. et al. (2020) recently reviewed ML and DL scoring functions for structure-based lead optimization developed between 2015 and 2019, but several new DL SFs have been developed and published in the last 2 years. Another example is the review of Shen et al. (2019), where only one GNN-based scoring function—PotentialNet (Feinberg et al., 2018)—was identified; most GNN scoring functions in Table 2 are from 2020 and later.

**TABLE 2:** Non-exhaustive list of deep learning architectures for protein-ligand binding affinity prediction and their performance on the CASF-2016 scoring benchmark (if available). MLPs are included regardless of the number of hidden layers. Some methods are described in multiple publications and the ones referenced in this table are the ones where the model has been evaluated on the PDBbind Core set 2016/CASF-2016 set (or the original publication, if this evaluation is not available). The best result (the highest Pearson’s r) is reported. Different publications might use slightly different custom variations of the CASF-2016 benchmark and the overlap between training and test sets might be taken into account in different ways. We refer the reader to the original publications for details, but we also report the number, N, of systems in the test set to outline possible differences. RMSEs are expressed in pK units.

Model	References	Architecture	Pearson’s *r*	RMSE	*N*
—	Artemenko (2008)	MPL	—	—	—
NNScore 2.0	Durrant and McCammon (2011b)	MPL	—	—	—
BgN- & BsN-Score	Ashtawy and Mahapatra (2015)	MPL	—	—	—
DLscore	Hassan et al. (2018)	MPL	—	—	—
PLEC-NN	Wójcikowski et al. (2018)	MLP	0.82	—	290
Pair	Zhu et al. (2020)	MLP	0.75	1.44	285
AEScore	Meli et al. (2021)	MLP	0.83	1.22	285
TopologyNet	Cang and Wei (2017)	CNN	0.81	1.34	290
K_deep_	Jiménez et al. (2018)	CNN	0.82	1.27	290
Pafnucy	Stepniewska-Dziubinska et al. (2018)	CNN	0.78	1.42	290
1D2D-CNN	Cang et al. (2018)	CNN	0.85	1.21	290
DeepAtom	Li et al. (2019c)	CNN	0.81	1.32	290
OnionNet	Zheng et al. (2019)	CNN	0.82	1.28	290
Gnina	Francoeur et al. (2020)	CNN	0.80	1.37	280
RosENet	Hassan-Harrirou et al. (2020)	CNN	0.82	1.24	
AK-Score	Kwon et al. (2020)	CNN	0.81	—	285
LigityScore1D	Azzopardi and Ebejer (2021)	CNN	0.74	1.46	285
OnionNet-2	Wang et al. (2021d)	CNN	0.86	1.16	285
SE-OnionNet	Wang et al. (2021b)	CNN	0.83	—	285
ACNN	Gomes et al. (2017)	GNN	—	—	—
PotentialNet	Feinberg et al. (2018)	GNN	—	—	—
graphDelta	Karlov et al. (2020)	GNN	0.87	1.05	285
SIGN	Li et al. (2021c)	GNN	0.80	1.32	290
InteractionGraphNet	Jiang et al. (2021)	GNN	0.84	1.22	262
GraphBAR	Son and Kim (2021)	GNN	0.78	1.41	290
PLIG/GATNet	Moesser et al. (2022)	GNN	0.84	1.22	272
PIGNet	Moon et al. (2022)	GNN	0.76	—	283
—	Berishvili et al. (2019)	CNN/RNN	—	—	—
FAST	Jones et al. (2021)	CNN + GNN	0.81	1.31	290
BAPA	Seo et al. (2021)	CNN + ATT	0.82	1.30	285
PointTransformer	Wang et al. (2021c)	CNN + ATT	0.85	1.19	285

Table 2 reports the scoring performance of several deep learning SFs mostly based on MLPs, CNNs, and GNNs on the CASF-2016 benchmark (whenever available in the primary reference). Tables 3, 4 report the scoring performance (Pearson’s correlation coefficient) for the CSAR-NRC HiQ sets and the Astex Diverse Set for the same methods outlined in Table 2. The significantly lower number of methods tested on the CSAR-NRC HiQ sets and the Astex Diverse Set shows that the CASF benchmark is the *de facto* standard for the assessment of novel ML and DL scoring functions. Going forward, it would be interesting to see the other benchmarks gaining more traction in order to obtain more information about scoring function performance.

**TABLE 3:** Performance of the models summarized in Table 2 on the CSAR-NRC HiQ scoring benchmark. We only report evaluation results from the original reference. RMSEs are expressed in *pK* units.

Model	References	Set 1 *r*	Set 1 RMSE	Set 2 *r*	Set 2 RMSE
K_deep_	Jiménez et al. (2018)	0.72	2.08	0.65	1.91
RosENet	Hassan-Harrirou et al. (2020)	0.83	1.78	0.80	1.44
OnionNet-2	Wang et al. (2021d)	0.89	1.50	0.87	1.21
graphDelta	Karlov et al. (2020)	0.74	1.59	0.71	1.52
GraphBAR	Son and Kim (2021)	0.75	1.59	0.65	1.56
PIGNet	Moon et al. (2022)	0.77	—	0.80	—
BAPA	Seo et al. (2021)	0.83	1.06	0.75	0.98

**TABLE 4:** Performance of the models summarized in Table 2 on the Astex Diverse Set scoring benchmark. We only report evaluation results from the original reference. RMSEs are expressed in *pK* units.

Model	References	Pearson’s *r*	RMSE
Pafnucy	Stepniewska-Dziubinska et al (2018)	0.57	1.37
DeepAtom	Li et al. (2019c)	0.77	1.03
RosENet	Hassan-Harrirou et al. (2020)	0.48	1.65

Despite the standardized benchmarks, some methods required the removal of some systems—leading to parametrization problems or outside the applicability domain—, but it is clear that most methods achieve similar performance on this benchmark. Additionally, the comparison between different methods on the same benchmark remains challenging due to possible differences in the training set—and the possible overlap between training and test sets. Finally, most methods are only tested on the CASF benchmark, despite other benchmark sets being widely available. These observations call for an in-depth comparison of the different methods trained and tested on exactly the same data sets, and using all available high-quality test sets.

The performance on CASF-2016 of the DL methods reviewed here is much higher than the performance of classical SFs on the same benchmark (Su et al., 2018). However, deep learning scoring functions do not always perform better or significantly better than scoring functions based on classical ML algorithms (Li H. et al., 2020). For example, it was shown that deep NNs and shallow regularized NNs perform similarly in QSAR applications when using the same set of descriptors (Winkler and Le 2016), and RF-based methods can achieve state-of-the-art performance when combined with suitable descriptors (Boyles et al., 2019). This is in stark contrast with other fields such as computer vision and natural language processing, where DL has quickly taken over classical ML algorithms. Additionally, while most ML and DL SFs for binding affinity prediction are trained and tested on crystal structures, their performance deteriorates when trained and tested on docked poses (Boyles et al., 2021), but it is worth noting that augmenting structure-based features obtained from docked structures with ligand-based features can recover the performance of structure-based models trained on crystal structures.

Another problem identified with ML and DL stricture-based SFs for binding affinity prediction is that while they perform significantly better than classical SFs for scoring (better correlation of the score with experimental binding affinities), they often perform poorly in virtual screening tasks (Gabel et al., 2014). Gabel et al. (2014) suggest that the development of novel ML and DL scoring function for binding affinity predictions should be accompanied by analysis of ligand pose sensitivity and enrichment capabilities in structure-based virtual screening. A more recent study by Shen et al. (2020a) confirms that ML scoring functions trained on PDBbind do not work well for virtual screening, especially on novel targets or targets with unconventional binding pockets. Multitask learning for binding affinity prediction and pose prediction trained using docked poses instead of crystallographic structures is effective to increase pose sensitivity in the context of CNN scoring functions (Francoeur et al., 2020). In the context of virtual screening, data augmentation techniques can also increase pose sensitivity by forcing the model to rely less on ligand information (Scantlebury et al., 2020).

It is well known that the maximum achievable performance of ML and DL models for binding affinity predictions is limited by experimental errors and uncertainties (Kramer et al., 2012). This explains the similar performance of the best performing models on CASF-2016, which are likely close to the theoretical limit. Ventures like the Critical Assessment of Computational Hit-finding Experiments (CACHE) (Müller et al., 2022) will play an important role to validate computational methods in the future and generate a larger corpus of very high-quality data.

Going forward, it is important to evaluate ML and DL scoring functions as part of the docking pipeline. Most SFs discussed here are applied as a post-processing step of docking—or they are only applied to crystal structures—and only a few SFs seem to have been incorporated into readily available docking software. One such example is GNINA, where the CNN scoring function can be employed within the docking pipeline to re-score or locally optimize the ligand poses after fast Monte Carlo search (McNutt et al., 2021).

In this review, we have focussed mainly on methods for the prediction of protein-ligand binding affinity, and scoring functions evaluated on scoring tasks. However, ranking different compounds against the same target of interest is extremely useful in drug discovery applications. This is the case for lead optimization, where a lead compound against the target of interest has been identified and the goal is to increase potency while improving pharmacokinetic and pharmacodynamic properties. With binding affinity predictions computing such rankings is trivial. However, it remains unclear if the performance of ML and DL methods developed for scoring work equally well for ranking, especially in real drug discovery applications. For example some methods trained to predict binding affinities performed poorly on the different task of predicting the differences in binding affinity upon protein mutation (Aldeghi et al., 2018b). DL methods specifically designed for ranking—computing relative binding affinities—have been developed (Jiménez-Luna et al., 2019) and are an active area of research (McNutt and Koes 2022).

Given that the performance of a DL SFs varies widely from the target under consideration (Jiménez et al., 2018; Hassan-Harrirou et al., 2020; Meli et al., 2021), there is a lot of room for improvement in the development of target-specific scoring functions (Ross et al., 2013; Nogueira and Koch 2019). ML and DL algorithms are very good at exploiting similarities between inputs to perform predictions—as demonstrated by the performance drop when similarities between the training and test sets are removed (Boyles et al., 2019; Su et al., 2020)—and therefore family-specific scoring function will play an increasing role in early stages of drug discovery, when a particular target has been identified. However, it is still unclear if family-specific structure-based SFs consistently outperform ligand-based methods (Shen et al., 2020a).

Finally, given the ultimate goal of lowering the high attrition rate at later stages of drug discovery, the use of ADME/Tox predictions will also play an increasingly important role (Bhhatarai et al., 2019) alongside SFs to identify potent compounds against the target of interest and prioritize compounds for further experimental validation.

While the application of deep learning has not yet provided a step-changing improvement in the performance of binding affinity prediction compared to classical ML methods, further research into novel architectures, combined with the ever-increasing size and quality of data sets of protein-ligand complexes might change the tide in the future. Physics-based ML and DL will probably take over purely data-driven models in the long term, combining the best of both worlds. It is however important to remain realistic on the capabilities of DL SFs and it will be interesting to see how they actually perform in real-world drug discovery applications. Schneider et al. (2019) suggest a “curious but cautious approach” to the application of DL in the drug discovery process. XAI methods will certainly play a central role in the application of DL scoring functions to real drug discovery programs because knowing the reason behind a given prediction and understanding well the failure modes of the developed models will help to guide the next steps in the drug discovery process.
